# Dual-specificity MAP kinase phosphatases in health and disease^☆^

**DOI:** 10.1016/j.bbamcr.2018.09.002

**Published:** 2019-01

**Authors:** Ole-Morten Seternes, Andrew M. Kidger, Stephen M. Keyse

**Affiliations:** a Department of Pharmacy, UiT The Arctic University of Norway, N-9037 Tromsø, Norway; b Signalling Programme, The Babraham Institute, Babraham Research Campus, Cambridge CB22 3AT, England, UK; c Stress Response Laboratory, Jacqui Wood Cancer Centre, James Arrot Drive, Ninewells Hospital & Medical School, Dundee DD1 9SY, UK

**Keywords:** MAP kinase, MAP kinase phosphatase, Diabetes, Obesity, Neuropathology, Oncogenic signalling

## Abstract

It is well established that a family of dual-specificity MAP kinase phosphatases (MKPs) play key roles in the regulated dephosphorylation and inactivation of MAP kinase isoforms in mammalian cells and tissues. MKPs provide a mechanism of spatiotemporal feedback control of these key signalling pathways, but can also mediate crosstalk between distinct MAP kinase cascades and facilitate interactions between MAP kinase pathways and other key signalling modules. As our knowledge of the regulation, substrate specificity and catalytic mechanisms of MKPs has matured, more recent work using genetic models has revealed key physiological functions for MKPs and also uncovered potentially important roles in regulating the pathophysiological outcome of signalling with relevance to human diseases. These include cancer, diabetes, inflammatory and neurodegenerative disorders. It is hoped that this understanding will reveal novel therapeutic targets and biomarkers for disease, thus contributing to more effective diagnosis and treatment for these debilitating and often fatal conditions.

## Highlights

• A comprehensive review of the dual-specificity MAP kinase Phosphatases (MKPs)
• Focus is on MKPs in the regulation of MAPK signalling in health and disease.
• Covers roles of MKPs in inflammation, obesity/diabetes, cancer and neurodegeneration

## 1 Introduction

**Fig. 1:**
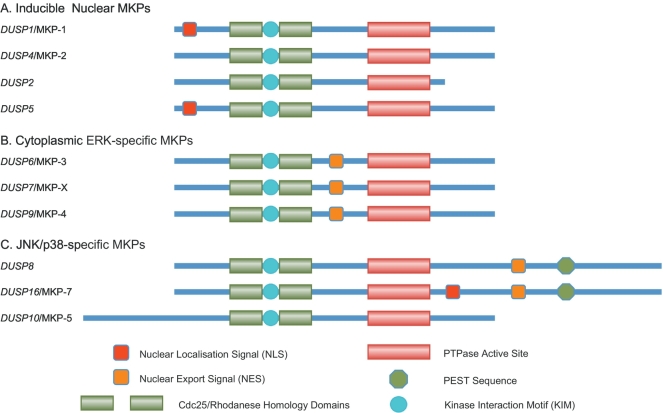
*Schematic showing the domain structures of the three groups of dual*-*specificity MAP kinase phosphatases*. A. The nuclear inducible MKPs. B. The cytoplasmic ERK-specific MKPs and C. the JNK/p38-specific MKPs. In addition to the amino-terminal non-catalytic domain and the PTPase active site, other key features and sequence motifs are indicated.

Mammalian dual-specificity MAP kinase (MAPK) phosphatases (MKPs) comprise a subfamily of 10 catalytically active enzymes with a conserved domain structure. This consists of an amino-terminal non-catalytic domain and a carboxyl-terminal catalytic domain. The former contains the kinase interaction motif (KIM), which determines the specific binding and thus substrate selectivity of the MKP for the different MAP kinase isoforms and can also contain nuclear localisation (NLS) or export (NES) signals, which determine the subcellular localisation of certain MKPs. The catalytic domain carries the highly conserved active site consensus sequence (HCX_5_R) that is characteristic of the larger protein tyrosine phosphatase (PTPase) superfamily. The regulation, structure, catalytic mechanism and substrate selectivity of the MKPs have been extensively reviewed [[1], [2], [3], [4], [5], [6]]. Briefly, the 10 enzymes can be divided into three subgroups based on amino acid sequence homology, subcellular localisation and substrate specificity. These are the inducible nuclear MKPs, comprising *DUSP1*/MKP-1, *DUSP2*, *DUSP4*/MKP-2 and *DUSP5*, the cytoplasmic, extracellular-signal regulated kinase (ERK) -specific MKPs *DUSP6*/MKP-3, *DUSP7*/MKP-X and *DUSP9*/MKP-4 and a group of three MKPs *DUSP8*, *DUSP10*/MKP-5 and *DUSP16*/MKP-7 that are found in both the cytoplasm and cell nucleus and are relatively selective in their ability to dephosphorylate the p38 and c-Jun amino terminal kinases (JNKs), having little or no activity towards the classical extracellular signal-regulated kinase (ERK) MAPKs (Fig. 1). Key features and characteristics of each of the 10 MKPs are also summarised (Table 1).

**Table 1:** Classification and properties of the mammalian dual specificity MAP kinase phosphatases.

Group	Gene/MKP	Subcellular localisation	Substrate specificity	Mouse models	References
Nuclear, inducible MKPs	*DUSP1*/MKP-1	Nuclear	JNK, p38 > ERK	UC, C	[16,56]
	*DUSP2*	Nuclear	ERK, JNK	UC	[86,87]
	*DUSP4*/MKP-2	Nuclear	ERK, JNK > p38	UC	[97]
	*DUSP5*	Nuclear	ERK	UC	[128]
Cytoplasmic ERK-selective MKPs	*DUSP6*/MKP-3	Cytoplasmic	ERK	UC	[149]
	*DUSP7*/MKP-X	Cytoplasmic	ERK	UC*	IMPC
	*DUSP9*/MKP-4	Cytoplasmic	ERK > p38	UC^**ǂ**^, C	[175,183]
JNK/p38-selective MKPs	*DUSP8*	Cytoplasmic/nuclear	JNK, p38	N/A	
	*DUSP10*/MKP-5	Cytoplasmic/nuclear	JNK, p38	UC	[185]
	*DUSP16*/MKP-7	Cytoplasmic/nuclear	JNK, p38	UC^**ǂ**^	[195,196]

UC, unconditional. C, conditional. *, infertile. ǂ, embryonic lethal. IMPC, International Mouse Phenotyping Consortium.

Our understanding of the physiological and pathophysiological roles for the MKPs has largely been driven by the generation of genetically engineered mouse (GEM) models in which individual MKPs have been deleted, either unconditionally, or in a tissue specific manner. This work, combined with studies in other model organisms, cell lines and observations in human cells and tissues has gradually revealed that MKPs play fundamental roles in the regulation of signalling events associated with normal development and homeostasis, but can also modulate a wide range of pathophysiological signalling outcomes with relevance to human disease. In this review we will detail the current level of understanding for each of the MKPs in turn, highlighting recent advances and future perspectives in the field.

## 2 The inducible nuclear MKPs

### 2.1 *DUSP1*/MKP-1

*DUSP1*/MKP-1 was the first of the dual-specificity MKPs to be characterised and was initially discovered as a growth factor or stress-inducible gene encoding a nuclear protein with homology to VH1, the prototypic dual-specificity protein phosphatase encoded by vaccinia virus [7,8]. Initially characterised as a phosphatase able to specifically dephosphorylate the threonine and tyrosine residues of the signature T-*E*-Y motif within the activation loop of the classical MAPK ERK2 *in vitro* and *in vivo* [9,10] it was later realised that *DUSP1*/MKP-1 was capable of dephosphorylating all three major classes of MAPK with a distinct preference for the JNK isoforms followed by p38α and ERK1/2 MAPKs [[11], [12], [13], [14], [15]]. *DUSP1*/MKP-1 was also the first gene encoding an MKP to be deleted in the mouse, where no phenotype was initially reported with respect to development, fertility or lifespan and no evidence for deregulated ERK signalling was found in *DUSP1*^−/−^ mouse embryo fibroblasts (MEFs) [16]. The failure to detect changes in MAPK activity in cells lacking *DUSP1*/MKP-1 was probably due to an initial focus on the ERK1/2 pathway. Subsequent work in MEFs clearly showed that loss of *DUSP1*/MKP-1 caused a significant increase in the activities of the stress-induced JNK and p38 MAPKs and revealed that MEFs lacking MKP-1 are acutely sensitive to JNK-mediated apoptosis in response to a wide variety of cellular stresses including UV-radiation, ionising radiation, hydrogen peroxide, anisomycin and cisplatin [[17], [18], [19], [20], [21]]. Further experiments conducted using *DUSP1*^−/−^ mice quickly led to the realisation that this phosphatase regulates a number of physiological and pathophysiological processes including immunity, metabolic homeostasis, cellular responses to anticancer drugs, muscle regeneration, and neuronal function.

#### 2.1.1 *DUSP1*/MKP-1 in innate and adaptive immunity

**Fig. 2:**
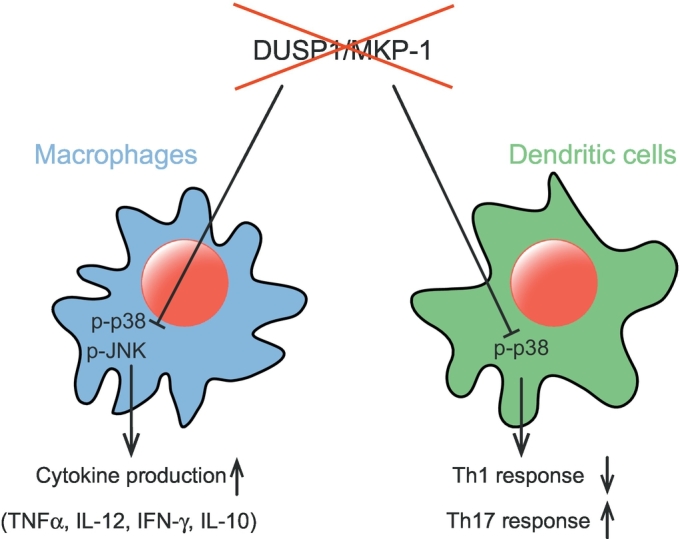
*DUSP1/MKP*-*1 in innate immunity*. Schematic showing the regulation of MAP kinase activities in cells of the innate immune system by *DUSP1*/MKP-1 and the consequences of genetic deletion of this MKP on the physiological responses of these cell populations. For details see text.

Given the wide range of roles that MAPKs perform in the development and function of cells of the immune system [[22], [23], [24]] it was perhaps no surprise that amongst the first phenotypes detected in *DUSP1*^−/−^ mice was a failure to regulate stress-activated JNK and p38 signalling in macrophages and dendritic cells (Fig. 2: Table 2). These cells are key mediators of the innate immune response in which the p38 and JNK MAPKs lie downstream of the toll-like receptors (TLRs), which are activated by a wide variety of pathogen-derived stimuli and act to regulate the expression of both pro and anti-inflammatory cytokines and chemokines [22]. Several groups demonstrated that loss of *DUSP1*/MKP-1 led to elevated JNK and p38 activities in macrophages exposed to the bacterial endotoxin lipopolysaccharide (LPS) [[25], [26], [27], [28]]. This led to an initial increase in the expression of pro-inflammatory cytokines such as tumour necrosis factor alpha (TNFα), interleukin-6 (IL-6), interleukin-12 (IL-12) and interferon-gamma (IFN-γ) while, at later times, levels of the anti-inflammatory mediator interleukin-10 (IL-10) were increased [25]. These cellular effects were accompanied by pathological changes such as inflammatory tissue infiltration, hypotension and multiple organ failure, all of which are markers of the severe septic shock and increased mortality observed in LPS-injected *DUSP1*^−/−^ mice when compared to wild type controls.

**Table 2:** Immunological phenotypes of MKP KO mice.

Group	Gene/MKP	Immunological phenotypes of MKP KO mice	References
Nuclear, inducible MKPs	*DUSP1*/MKP-1	Increased pro-inflammatory cytokine production & innate immune response LPS challenge.	[25]
		Impaired resolution of inflammation.	[30]
		Decreased adaptive immune response & viral clearance.	[33]
		Protection from autoimmune encephalitis (EAE).	[33]
		Increased sensitivity to bacterial infections.	[35]
		Exacerbates inflammatory phenotypes including: colitis, anaphylaxis and psoriasis.	[[40], [41], [42]]
	*DUSP2*	Protection from experimentally-induced arthritis.	[86]
		Decreased macrophage cytokine expression & mast cell survival.	[86]
		Increased susceptibility to DSS-induced model of intestinal inflammation.	[87]
		Altered T-cell balance, *via* the promotion of Th17 differentiation and inhibition of Treg generation.	[87]
	*DUSP4*/MKP-2	Increased susceptibility to *Leishmania mexicana*, *Leishmania donovani & Toxoplasma gondii* infection.	[[97], [98], [99]]
		Resistant to LPS-induced endotoxic shock.	[100]
		Increased CD4+ T-cell proliferation.	[101]
		Protection from autoimmune encephalitis (EAE).	[102]
	*DUSP5*	Negatively regulates Il-33 mediated eosinophil survival.	[119]
		Resistant to helminth infection, due to enhanced eosinophil activity.	[119]
		Regulates CD8+ populations in response to LCMV infection.	[120]
Cytoplasmic ERK-selective MKPs	*DUSP6*/MKP-3	Exacerbates intestinal colitis.	[150]
		Decreased CD4+ T-cell proliferation, altered T-cell polarisation & impaired Treg function.	[150]
	*DUSP7*/MKP-X	N/A	
	*DUSP9*/MKP-4	N/A	
JNK/p38-selective MKPs	*DUSP8*	N/A	
	*DUSP10*/MKP-5	Impaired T cell expansion, but enhanced priming of T-cells by APCs.	[185]
		Protection from autoimmune encephalitis (EAE).	[185]
		Increased cytokine and ROS production in macrophages, neutrophils and T cells.	[187]
		Protection from DSS-induced intestinal inflammation.	[191]
	*DUSP16*/MKP-7	Impaired GM-CSF-driven proliferation of bone marrow progenitors.	[195]
		Increased CD4+ T-cell proliferation & a reduced Th17 cell population.	[196]
		Protection from autoimmune encephalitis (EAE).	[196]

LCMV, lymphocytic choriomeningitis virus. DSS, dextran sodium sulfate.

With respect to the above changes in cytokine expression, the regulation of gene transcription by MAPK-regulated transcription factors such as activator protein-1 (AP1), activating transcription factor 1 (ATF-1) and cAMP response element binding protein (CREB) by *DUSP1*/MKP-1 was an early focus [25,29]. However, a major mechanism by which cytokine expression is controlled is *via* changes in mRNA stability and recent studies have revealed that *DUSP1*/MKP-1 modulates cytokine mRNA levels by suppressing the p38 driven MAPK-activated protein kinase 2 (MK2)-dependent phosphorylation of the mRNA destabilising protein tristetraprolin (TTP) [30]. TTP, which recognizes adenosine/uridine-rich elements (AREs) in the 3′ untranslated regions (UTRs) of cytokine mRNAs and recruits components of the cellular mRNA degradation machinery is phosphorylated by MK-2 on two sites (Ser52 and 178), which leads to both inactivation and stabilisation of TTP [31]. Thus, loss of *DUSP1*/MKP-1, by promoting p38-MK2-driven phosphorylation of TTP, favours TTP inactivation and cytokine mRNA stabilisation. In an elegant series of experiments, Smallie et al., combined deletion of *DUSP1*/MKP-1 with a homozygous knock-in mutant in which the MK2-dependent phosphorylation sites within TTP are ablated and demonstrated that in bone marrow-derived macrophages (BMDMs) derived from the double mutant mice the elevated cytokine mRNA and protein levels seen on deletion of *DUSP1*/MKP-1 alone was largely prevented. A similar reversal in the elevated serum levels of cytokines seen in LPS-injected *DUSP1*^−/−^ mice was also observed in the double mutant animals and microarray experiments performed using LPS-treated BMDMs, indicate that *DUSP1*/MKP-1 regulates more than half of the genome-wide response to LPS, either wholly or partly *via* the phosphorylation of TTP [30]. A similar approach revealed that production of interferon beta (IFNβ) in response to TLR activation is also mediated in part by *DUSP1*/MKP-1-mediated regulation of TTP, but that in the early phase of the response *DUSP1*/MKP-1 regulates IFNβ transcription *via* JNK-mediated phosphorylation of c-jun, which binds to the IFNβ promoter [32].

Taken together, this work demonstrates that TLR mediated expression of *DUSP1*/MKP-1 is a key component of a pathway, which acts through regulation of MAPK-dependent transcription factors and TTP to negatively regulate pathological inflammatory responses, to engage the “off phase” of macrophage-mediated responses to pro-inflammatory stimuli and promote the resolution of inflammation. As such, any defects in this pathway would be expected to impede the latter process and contribute to a range of chronic inflammatory diseases, making the *DUSP1*/MKP-1-p38-MK2 signalling axis a prime candidate for therapeutic intervention.

While innate immunity comprises an acute, non-specific response to foreign antigens, adaptive or acquired immunity is highly specific to a particular antigenic stimulus and comprises a network of specialized, immune cells and processes that either eliminate pathogens or prevent their growth. In addition, by generating immunological memory, this response also provides long-lasting immunity against infection, which is the basis of vaccination, while an abnormal or maladaptive response can result in autoimmune disease. The workhorses of the system are the B and T lymphocytes, which mediate humoral (antibody-mediated) immunity and cell-mediated (cytotoxic or effector cell-mediated) responses. Despite the key role for ERK signalling in thymocytes and the observation that *DUSP1*/MKP-1 is expressed at varying levels during T-cell development, mice lacking *DUSP1*/MKP-1 do not present with abnormalities in this process and the ratio of CD4^+^ to CD8^+^ T-cells following thymic maturation is in the normal range [33]. This possibly reflects either redundancy amongst ERK-specific phosphatases or the fact that ERK is not the preferred target for *DUSP1*/MKP-1.

However, in mature CD4+ T cells, loss of *DUSP1*/MKP-1 seems to impact T cell function with decreased activation and proliferation following exposure to phorbol 12-myristate 13-acetate (PMA) and ionomycin and increased levels of JNK signalling [33]. Furthermore, both CD4+ and CD8+ T cells lacking *DUSP1*/MKP-1 showed reduced proliferation and interleukin-2 (IL-2) production after exposure to anti-CD3 antibody to mimic T cell receptor activation, either alone or in combination with anti-CD28. This lack of proliferation correlated with a failure to accumulate nuclear factor of activated T cells c1 (NFATc1) in the cell nucleus and, as this process is negatively regulated by JNK signalling, most probably reflects a failure to restrain JNK activity in these cells [33]. Consistent with this, re-stimulation of activated *DUSP1*^−/−^ CD4+ T cells with anti-CD3 also caused an increase in JNK-dependent activation-induced cell death (AICD). The differentiation of effector T cell lineages was also affected by deletion of *DUSP1*/MKP-1 with naïve *DUSP1*^−/−^ CD4+ T cells showing deficits in effector cytokine producing type-1 helper (Th1) and pro-inflammatory type-17 helper (Th17) cell differentiation and function, while in naïve CD8^+^ T cells *DUSP1*/MKP-1 deficiency resulted in lower production of the CD8^+^ T cell effector cytokines IFN-γ and TNFα [33]. Finally, *DUSP1*/MKP-1was found to be required for anti-influenza T cell responses in infected mice with infected *DUSP1*^−/−^ animals showing defective influenza virus-specific CD4^+^ and CD8^+^ T cell responses and clear signs of impaired viral clearance. In contrast, mice lacking *DUSP1*/MKP-1 were protected from experimentally induced autoimmune encephalitis (EAE) following injection of myelin oligodendrocyte glycoprotein peptide (MOG_35–55_). This resulted from an intrinsic defect in MKP-1 KO CD4^+^ T cells, which showed reduced production of IL-17 and IFNγ and demonstrates a key role for *DUSP1*/MKP-1 in mediating autoreactive CD4^+^ T cell responses in vivo [33].

As well as performing key functions in innate immunity, dendritic cells form a bridge between innate and adaptive immune responses by acting as antigen presenting cells for the priming of both CD4+ T helper (Th) and CD8+ cytotoxic T lymphocytes (Tc) [34]. As well as affecting the function of these T cell subsets, it turns out that *DUSP1*/MKP-1 also plays a key role in facilitating this crosstalk. Huang et al. [35] used a model in which the immune system in lethally irradiated mice was reconstituted with a mix of bone marrow from *DUSP1*^−/−^/Rag1^−/−^ and WT mice (5:1 ratio) and compared with mice reconstituted using *DUSP1*^+/+^/Rag1^−/−^ and WT (5:1 ratio) bone marrow. In both cohorts the T cells were derived from the WT marrow (Rag1^−/−^ bone marrow cannot generate mature T and B cells) and thus expressed *DUSP1*/MKP-1 while the cells of the innate immune system were either null or WT for *DUSP1*/MKP-1. Using two mouse infection models, the *Listeria monocytogenes* (Th1 biased model) and *Candida albicans* (Th17 biased model), they found that dendritic cells lacking *DUSP1*/MKP-1 exhibited reduced IL-12 production and attenuated IFNγ expression and Th1 responses. In contrast, the production of IL-6 by dendritic cells lacking *DUSP1*/MKP-1 was enhanced and this resulted in an exaggerated Th17 response. In addition, *DUSP1*/MKP-1 suppressed the release of transforming growth factor β2 (TGFβ2) by dendritic cells, thus inhibiting the development of inducible regulatory T cells (Treg). At the biochemical level, these altered responses were mediated by increased p38 MAPK activity in dendritic cells lacking *DUSP1*/MKP-1. In conclusion this work clearly shows that the activity of *DUSP1*/MKP-1 in the dendritic cells of the innate immune system is a critical regulator of signals that dictate the course of adaptive immune responses at the immunological synapse [35].

One interesting observation arising from these studies of *DUSP1*/MKP-1 in innate and adaptive immunity is that whereas *DUSP1*/MKP-1 mainly targets p38 MAPK in macrophages and dendritic cells, the T cell effects of *DUSP1*/MKP-1 loss seem to be mediated predominantly by increased JNK activity. This suggests that there is cell type specificity with respect to *DUSP1*/MKP-1 activity towards different MAPK isoforms. The mechanism by which this might be achieved is unclear, but may be related to post-translational modification. *DUSP1*/MKP-1 is phosphorylated and this is known to modulate its stability [36,37]. More recently, it was shown that p300 histone acetylase-mediated acetylation of lysine 57, which lies just C-terminal of the KIM within the amino terminal domain of *DUSP1*/MKP-1, reinforces its interaction with and ability to dephosphorylate p38 MAPK [38]. This can be opposed by a subset of specific histone deacetylases (HDACs 1–3) in mouse macrophages [39], suggesting one possible mechanism by which the canonical substrate selectivity of *DUSP1*/MKP-1 might be regulated in a cell type specific manner.

Finally, given its key role as a critical regulator of innate and adaptive immunity (Table 2), loss of *DUSP1*/MKP-1 was also found to exacerbate a range of inflammatory phenotypes in mouse models including experimental colitis [40], anaphylaxis [41] and psoriasis [42], However, for reasons as yet unclear, loss of *DUSP1*/MKP-1 did not sensitize mice to the development of spontaneous age-dependent osteoarthritis, despite the involvement of an inflammatory process and mediators such as TNFα and interleukin-1β in this disease [43]. *DUSP1*/MKP-1 is also directly targeted by a range of immune modulators. Enhanced expression of *DUSP1*/MKP-1 underpins, at least in part, the anti-inflammatory activity of glucocorticoids [44,45] and is also observed in response to vitamin D [46] and transforming growth factor-beta (TGFβ) [47] both of which are anti-inflammatory. In contrast, pro-inflammatory stimuli such as IFN-γ and interleukin-17A (IL-17A) suppress *DUSP1*/MKP-1 expression and thus increase signalling through the p38 and JNK MAPK pathways [48,49].

#### 2.1.2 *DUSP1*/MKP-1 in metabolic homeostasis

**Fig. 3:**
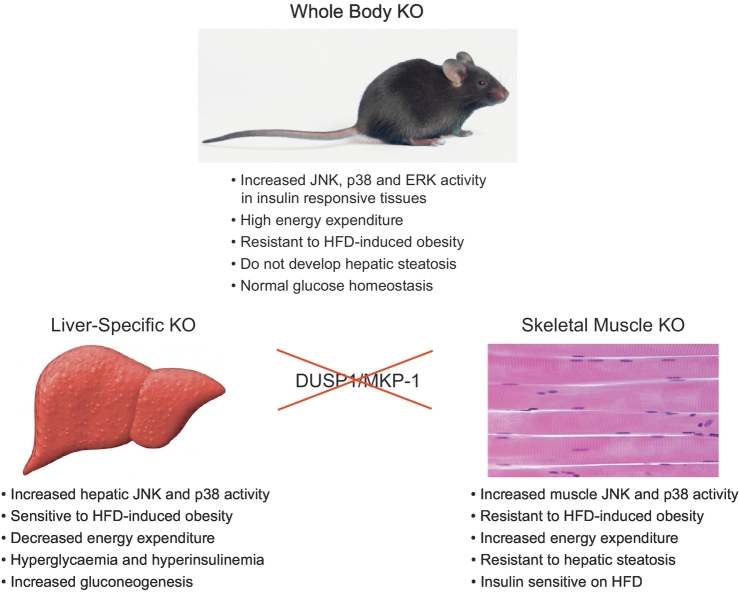
*DUSP1/MKP*-*1 in metabolic homeostasis*. Schematic showing the effects of either unconditional, or tissue-specific, deletion of *DUSP1*/MKP-1 on metabolic homeostasis. For details see text.

The first indication that *DUSP1*/MKP-1 might play a role in regulating metabolic homeostasis came with the finding that *DUSP1*^−/−^ mice were resistant to diet-induced obesity (Fig. 3) and that this reflected a higher level of energy expenditure, but not overall activity in the null mice [50]. Surprisingly, despite remaining lean on a high-fat diet (HFD), *DUSP1*^−/−^ mice did become glucose intolerant (as would wild type animals), while still being protected from hepatic steatosis. This phenotype correlated with increased levels of JNK, p38 and ERK activity in insulin responsive tissues. However, *DUSP1*^−/−^ mice did not show abnormalities in insulin signalling or glucose homeostasis, despite an established role for JNK signalling in promoting insulin resistance [51]. This apparent contradiction was possibly due to the finding that loss of *DUSP1*/MKP-1 affects nuclear rather than cytoplasmic JNK activity and that the latter is responsible for JNK-dependent abnormalities in the response to insulin [50].

Subsequent work has revealed mechanistic aspects of the *DUSP1*^−/−^ metabolic phenotype. Firstly, mice lacking *DUSP1*/MKP-1 are protected from the loss of oxidative (slow-twitch) myofibers in skeletal muscle. The overall effect of this is to favour oxidative over glycolytic metabolism and, because the latter consumes less energy, to protect against diet-induced obesity [52]. This effect seems to be secondary to increased p38 MAPK-mediated phosphorylation and stabilisation of peroxisome proliferator-activated receptor-gamma coactivator 1α (PGC-1α) thus increasing its activity as a regulator of mitochondrial biogenesis and energy expenditure. Experiments in which grossly obese leptin-resistant (db/db) mice were crossed with *DUSP1*^−/−^ animals revealed that loss of *DUSP1*/MKP-1 protected against hepatic steatosis. By increasing MAPK-dependent phosphorylation of peroxisome proliferator-activated receptor-γ (PPARγ) at a site (Ser112) that negatively regulates its activity, loss of *DUSP1*/MKP-1 prevents the PPARγ-dependent expression of lipogenic genes, thus reducing lipid droplet formation in hepatocytes [53].

While this work sheds some light of the functions of *DUSP1*/MKP-1 in metabolic homeostasis a severe limitation of these studies is the use of a whole body *DUSP1*/MKP-1 knockout. Metabolic control is complex and subject to both central and peripheral regulation [54]. Furthermore, diet-induced obesity has an inflammatory component [55] and the role(s) of *DUSP1*/MKP-1 in regulating immune responses may also be a confounding factor. To begin to address this, a conditional *DUSP1*^fl/fl^ mouse has now been employed to study the metabolic effects of *DUSP1*/MKP-1 deletion in specific tissues.

Liver specific knockout of *DUSP1*/MKP-1 (MKP-1-LKO) using albumin-Cre (Alb-Cre) resulted in increased hepatic JNK and p38 activation. However, unlike *DUSP1*^−/−^ mice MKP-1-LKO animals exhibited increased adiposity, fasting hyperglycaemia and hyperinsulinemia on a normal chow diet, indicating that hepatic *DUSP1*/MKP-1 regulates glucose homeostasis (Fig. 3). This was confirmed in subsequent experiments using hyperinsulinemic-euglycemic clamps, which demonstrated that MKP-1-LKO mice were hyperglycaemic, glucose intolerant and develop hepatic insulin resistance [56]. While DUSP1^−/−^ mice were resistant to HFD-induced obesity MKP-1-LKO mice were more susceptible, but were still protected against hepatic steatosis. Furthermore, unlike *DUSP1*^−/−^ mice, they showed *decreased* energy expenditure [56]. Mechanistically, the effects of *DUSP1*/MKP-1 deletion on glucose metabolism were found to be secondary to increased hepatic p38 and JNK mediated transcription of gluconeogenic genes, increased p38-dependent phosphorylation of cyclic AMP responsive element binding protein (CREB), which promotes gluconeogenesis through PGC1/PPARγ and decreased activation of Signal Transducer and Activator of Transcription 3 (STAT3), a negative regulator of gluconeogenesis [56]. The latter effect is probably an indirect result of the lower circulating levels of IL-6 in MKP-1-LKO mice, as this metabolic cytokine is a potent inducer of Janus kinase (JAK)-STAT signalling. Finally, the decreased energy expenditure observed in MKP-1-LKO mice may be related to reduced levels of IL-6 and fibroblast growth factor 21 (FGF21). Both factors promote energy expenditure, insulin sensitivity, fatty acid oxidation, and weight loss and their reduction would be expected to impair skeletal muscle oxidative capacity and thus increase susceptibility to diet-induced obesity [56].

Skeletal muscle plays a major role in the regulation of glucose metabolism and metabolic homeostasis. Following on from the liver-specific deletion of *DUSP1*/MKP-1, the effects of skeletal muscle specific loss of this phosphatase (MKP-1-MKO), using human α-skeletal actin (HSA-Cre), have now been studied. MKP-1-MKO mice show increased levels of p38 and JNK signalling in skeletal muscle and are significantly protected from HFD-induced weight gain (Fig. 3). As was the case in the MKP1^−/−^ mice, the failure to gain weight was secondary to enhanced energy expenditure when compared with MKP-1^fl/fl^ controls and no differences in either food intake, or activity between the two genotypes was observed [57]. Interestingly, MKP-1-MKO mice were also resistant to hepatic steatosis, which was consistent with lower levels of hepatic PPARγ and sterol regulatory element-binding protein 1c (SREBP-1c) expression. However, no changes in either p38 or JNK activity were detected in liver tissue. Glucose (GTT) and insulin tolerance (ITT) tests revealed that MKP-1MKO mice on a HFD produced lower levels of circulating insulin and were insulin sensitive, indicating that they are protected from the development of insulin resistance. Biochemically, an unexpected role for increased PI3-kinase-Akt signalling resulting from microRNA-21 (miR-21) dependent down-regulation of phosphatase and tensin homolog (PTEN) in MPK-1-MKO was uncovered and this could contribute to the increased insulin sensitivity observed in MKP-1-MKO mice. Finally, consistent with the results of whole body deletion of *DUSP1*/MKP-1, the increased energy expenditure observed in MKP-1-MKO mice was secondary to an increase in the proportion of oxidative myofibers and was reflected in enhanced oxidative capacity and mitochondrial function in skeletal muscle [57].

Taken together, these results begin to unravel some of the complexity and tissue specific interplay of *DUSP1*/MKP-1 action in the regulation of metabolic homeostasis (Fig. 3; Table 3) and also emphasise the importance of compartmentalised nuclear regulation of p38 and JNK activities in mediating the phenotypes observed. The observation that *DUSP1*/MKP-1 is up-regulated in insulin-responsive tissues in response to a HFD in mice and also in obese humans indicates that it forms part of a key stress response that leads to decreased energy expenditure in skeletal muscle, thus contributing to weight gain and may also mediate at least some of the adverse consequences of this disease, including abnormalities in glucose metabolism and hepatosteatosis. The further use of conditional *DUSP1*/MKP-1 ablation will reveal the relative importance of MAPK regulation in distinct tissues by this phosphatase in energy homeostasis and, from the information gathered so far, MKP-1/*DUSP1* continues to be a potential pharmacological target for the treatment of metabolic disease.

**Table 3:** The involvement of MKPs in metabolic homeostasis.

Group	Gene/MKP	Evidence implicating functions for MKPs in metabolic homeostasis	References
Nuclear, inducible MKPs	*DUSP1*/MKP-1	**Whole Body KO:**	
		Resistant to diet-induced obesity.	[50]
		Protected from the loss of oxidative myofibers in skeletal muscle.	[52]
		Protected against hepatic steatosis.	[53]
		Increased energy expenditure.	[52]
		**Liver-specific KO:**	
		Glucose intolerant & susceptible to diet-induced obesity.	[56]
		Protected against hepatic steatosis.	[56]
		Decreased energy expenditure.	[56]
		**Skeletal muscle specific KO:**	
		Resistant to diet-induced obesity.	[57]
		Increased energy expenditure & oxidative myofibers.	[57]
	*DUSP2*	N/A	
	*DUSP4*/MKP-2	N/A	
	*DUSP5*	N/A	
Cytoplasmic ERK-selective MKPs	*DUSP6*/MKP-3	*DUSP6*/MKP-3 KO mice are resistant to high fat diet (HFD)-induced obesity & hepatosteatosis.	[155,156]
	*DUSP7*/MKP-X	N/A	
	*DUSP9*/MKP-4	*DUSP9* is implicated in regulation of the response to insulin and GWAS studies have identified *DUSP9* as a susceptibility locus for the development of Type-2 diabetes, in multiple ethnicities.	[179,180,182]
		Liver-specific loss of DUSP9/MKP-4 sensitises mice to HFD-induced obesity, hepatosteatosis, liver fibrosis and inflammation.	[181]
JNK/p38-selective MKPs	*DUSP8*	N/A	
	*DUSP10*/MKP-5	N/A	
	*DUSP16*/MKP-7	N/A	

#### 2.1.3 *DUSP1*/MKP-1 in cancer

Given the central importance of deregulated MAPK signalling in the initiation and progression of human cancers it is no great surprise that the involvement of MKPs in regulating various aspects of the cancer phenotype has been of widespread interest [[58], [59], [60]]. Disappointingly, given that *DUSP1*/MKP-1 was both the first MKP to be discovered and also the first to be deleted from the mouse genome, there are currently no published studies in which *DUSP1*/MKP-1 has been directly implicated in either tumour initiation or progression. It is hoped that the recent development of the conditional *DUSP1*/MKP-1 mouse (see 2.1.2.) will facilitate definitive experiments, particularly as this model avoids the potentially confounding effects of the immune and inflammatory abnormalities seen when *DUSP1*/MKP-1 is knocked out globally.

In contrast, over the 25 or so years since *DUSP1*/MKP-1 and its role in regulating MAP kinase signalling were discovered, there have been numerous publications reporting either increased or reduced expression of *DUSP1*/MKP-1 in a wide range of human tumours including breast, pancreas, gastric, ovary, lung, skin and prostate (Table 4). In addition, a number of studies have relied on ectopic overexpression of *DUSP1*/MKP-1 in normal and cancer cell lines to study its possible role in modulating oncogenic signalling. These studies have been extensively reviewed elsewhere [60] and as they have often yielded equivocal or even contradictory information regarding the role of *DUSP1*/MKP-1 in cancer, it is not proposed to list or discuss them further here.

**Table 4:** The involvement of MKPs in cancer.

Group	Gene/MKP	Cancer-related phenotypes	References
Nuclear, inducible MKPs	*DUSP1*/MKP-1	Reduced or increased expression noted in a number of tumour types and cancer cell lines.	[[58], [59], [60]]
		Elevated expression implicated in chemoresistance to a variety of anti-cancer drugs including cisplatin, gemcitabine, doxyrubicin, taxanes and intrinsic resistance to tyrosine kinase inhibitors.	[[63], [64], [65],[67], [68], [69], [70]]
	*DUSP2*	Down-regulation in a variety of solid tumours and in acute myeloid leukemia.	[[88], [89], [90]]
		Frequently mutated in Diffuse Large B-Cell lymphoma (DBCL).	[91,92]
	*DUSP4*/MKP-2	Reduced or increased expression noted in a number of tumour types and cancer cell lines.	[[58], [59], [60],^104^]
		Often epigenetically silenced in DBCL. Lack of expression is a negative prognostic factor.	[105]
		Implicated in resistance to doxyrubicin in gastric cancers and to Trastuzumab (anti-Her2 antibody) in Her2+ breast cancers.	[106,107]
	*DUSP5*	Expression often elevated in cancer cell lines harbouring mutant Ras of Raf oncogenes.	[[123], [124], [125]]
		Epigenetic silencing in gastric and colorectal cancers.	[126,127]
		Genetic deletion sensitises mice to DMBA/TPA-induced skin carcinogenesis.	[128]
		Oncogene dependent effects of *DUSP5* deletion on cell growth and transformation of mouse embryo fibroblasts.	[131]
Cytoplasmic ERK-selective MKPs	*DUSP6*/MKP-3	Expression often elevated in cancer cell lines harbouring mutant Ras of Raf oncogenes.	[125,158,159]
		Expression initially elevated and then lost during progression of mutant Kras-driven pancreatic ductal adenocarcinoma and lung cancers.	[[160], [161], [162], [163]]
		Positive correlation between *DUSP6*/MKP-3 expression and transformation by BCR-Abl and mutant Nras in a murine model of acute lymphobastic leukemia (ALL).	[164]
		Loss of *DUSP6*/MKP-3 reported to be synthetic lethal with mutant Braf in melanoma cell lines.	[165]
		Up-regulated in human gliobastoma and papillary thyroid carcinoma where it is associated with a pro-oncogenic function	[167,168]
	*DUSP7*/MKP-X	N/A	
	*DUSP9*/MKP-4	N/A	
JNK/p38-selective MKPs	*DUSP8*	N/A	
	*DUSP10*/MKP-5	N/A	
	*DUSP16*/MKP-7	N/A	

One aspect of cancer biology in which *DUSP1*/MKP-1 does appear to play an important role is in the response of normal and cancer cells to a range of chemical and physical insults including modalities used in cancer chemotherapy (Table 4). Soon after it became clear that the p38 and JNK MAPKs were the preferred substrates for *DUSP1*/MKP-1, it was observed that the overexpression of this phosphatase enhanced cellular resistance to both UV-radiation and the chemotherapeutic drug cisplatin and that this was related to the suppression of JNK-mediated apoptosis [61,62]. That *DUSP1*/MKP-1 played a crucial role in modulating sensitivity to these insults was confirmed when MEFs derived from *DUSP1*^−/−^ mice were found to be sensitive to UV-radiation, cisplatin, hydrogen peroxide and anisomycin [[17], [18], [19], [20]]. In normal cells, *DUSP1*/MKP-1 expression is induced by UV and cisplatin *via* activation of p38 MAPK, whereas it is the suppression of JNK activity by *DUSP1*/MKP-1 that modulates cell death. This indicates that *DUSP1*/MKP-1 mediated crosstalk between these two distinct MAPK pathways regulates cellular sensitivity [20].

Thus it is likely that elevated expression of *DUSP1*/MKP-1 in tumours can mediate chemoresistance and this is supported by studies in non-small cell lung cancer (NSCLC) where overexpression of *DUSP1*/MKP-1 is observed and patients become resistant to treatment with cisplatin. In NSCLC cancer cell lines where *DUSP1*/MKP-1 was constitutively expressed, siRNA knockdown increased cisplatin sensitivity some 10 fold, reduced the growth of these cell lines in nude mice and rendered the resulting tumours cisplatin sensitive [63]. In lung cancer patients dexamethasone is also often co-administered with cisplatin to ameliorate the undesirable side effects of treatment. However, glucocorticoids are known to upregulate *DUSP1*/MKP-1 expression [64] and not surprisingly dexamethasone effectively suppressed cisplatin-induced apoptosis in a lung adenocarcinoma cell line indicating that *DUSP1*/MKP-1 plays a key role in this potentially undesirable drug-drug interaction [65]. The role of *DUSP1*/MKP-1 in mediating resistance to chemotherapy appears not to be restricted to cisplatin, as the ability of this phosphatase to inhibit JNK-mediated apoptosis has also been implicated in the resistance of pancreatic cancer cells to gemcitabine [66], multidrug resistance in glioblastoma [67], resistance to doxorubicin and taxanes in breast cancer [68] and resistance to the proteasome inhibitor bortezomib [69].

Finally, a recent paper has implicated *DUSP1*/MKP-1 in a growth factor-dependent pathway, which promotes intrinsic resistance to the tyrosine kinase inhibitors (TKI) used to treat chronic myeloid leukemias (CML). In mouse pro-B BaF3 cells engineered to express the breakpoint cluster region (BCR)-Abl tyrosine kinase fusion, which drives CML, Kesarwani et al. [70] found that *DUSP1*/MKP-1 along with FBJ osteosarcoma oncogene (Fos) were responsible for resistance to the Abl TKI inhibitor imatinib (Gleevec). Genetic deletion or pharmacological inhibition of Fos and *DUSP1*/MKP-1 eradicated minimal residual disease (MRD) in multiple *in vivo* models as well as in patient-derived mouse xenografts. Mechanistically, *DUSP1*/MKP-1 seems to influence TKI sensitivity *via* its ability to suppress p38 MAPK activity and modulate AP1-dependent transcriptional networks. The latter hypothesis is supported by the finding that SB202190, a specific p38 MAPK inhibitor, also conferred imatinib resistance. While these results are potentially exciting, some caution is necessary in the interpretation of the data. BCI (2-benzylidene-3-(cyclohexylamino)-1-Indanone hydrochloride), the “specific” *DUSP1* inhibitor used to treat mice and reverse disease in a retroviral bone marrow transduction transplantation leukemogenesis model is both highly toxic and relatively non-specific [71].

#### 2.1.4 *DUSP1*/MKP-1 function in other tissues

Given the key role that MAPK signalling plays in aspects of brain development and function it is unsurprising that MKPs have been implicated in the regulation of these processes. Indeed *DUSP1*/MKP-1 plays important roles in neural cell development, neuronal cell survival and death, glial cell function and events, which underpin learning and memory (reviewed in [72]). In terms of pathophysiology, an important observation was that *DUSP1*/MKP-1 levels were elevated in the hippocampal region of post-mortem brain from patients who had been diagnosed with major depressive disorder (MDD) [73]. MDD is characterised by chronic or episodic depression and carries a significant (2–7%) risk of suicide.

Duric et al. [73] found that *DUSP1*/MKP-1 was also elevated in the hippocampus of rats exposed to chronic unpredictable stress (CUS) an effect that was attenuated by treatment with the antidepressant drug fluoxetine (Prozac) a selective serotonin reuptake inhibitor. Furthermore, adenoviral-mediated expression of *DUSP1*/MKP-1 in the hippocampus caused anhedonia (an inability to experience pleasure), as assessed by a reduced preference for sucrose over water, and these animals displayed other surrogates of depressive behaviour or helplessness such as disturbed feeding and increased immobility in the forced swim test, all of which were also seen in the CUS exposed rats. Interestingly, all of the latter endpoints were suppressed in CUS exposed *DUSP1*^−/−^ mice when compared to wild type controls [73].

Mechanistically, these changes were associated with a reduction in phospho-ERK1/2 levels in CUS exposed wild type mice, which was not observed in *DUSP1*^−/−^ mice. A result, which led the authors to conclude that ERK was the relevant *DUSP1*/MKP-1 target. This finding is somewhat surprising in the light of our knowledge that JNK and p38, but not ERK, are the preferred substrates for this phosphatase [15] and also conflicts with a previous study in which a reduction in hippocampal phospho-JNK but not phospho-ERK was observed in rats exposed to CUS [74]. Finally, a recent study has identified similar changes in *DUSP1*/MKP-1 levels in the anterior cingulate cortex (ACC) of mice exposed to neurophathic pain and CUS, which were again reversed by fluoxetine [75]. While not shedding new light on the biochemical mechanisms involved, this latter study does implicate the regulation of MAPK signalling by *DUSP1*/MKP-1 in another brain region tightly associated with regulating mood-related functions.

With respect to neurodegenerative disorders, *DUSP1*/MKP-1 has been reported to mediate neuroprotective effects in both *in vitro* and *in vivo* models of Huntington's disease through its ability to suppress polyglutamine-expanded huntingtin-induced activation of c-Jun N-terminal kinases (JNKs) and p38 MAPKs [76]. Finally, by suppressing p38 MAPK activity, *DUSP1*/MKP-1 has been reported to protect dopaminergic neurons from the toxic effects of 6-hydroxydopamine (6-OHDA) suggesting that strategies aimed at either increasing MKP-1 expression or activity might be a viable strategy in the treatment of Parkinson's disease [77].

*DUSP1*/MKP-1 has also been implicated in muscle regeneration as *DUSP1*^−/−^ mice are impaired in their ability to recover from experimental muscle injury and, when crossed into a mouse model of Duchenne's muscular dystrophy (the *mdx* dystrophin null), they display exacerbated muscular dystrophinopathy [78]. Interestingly, this is exactly the reciprocal of the phenotype observed after deletion of *DUSP10*/MKP-5 (see Section 4.2.2) [79]. More recently, the study of *DUSP1*^−/−^/*DUSP10*^−/−^ double knockout (DKO) mice revealed a severe impairment in muscle regeneration. Satellite cells, the precursors of muscle cells, were less proliferative and DKO mice had increased inflammation at sites of injury suggesting that the positive regulation of myogenesis by *DUSP1*/MKP-1 is dominant over negative regulation by *DUSP10*/MKP-5 [80]. Despite the fact that they share common substrates in JNK and p38 it is clear that these two MKPs regulate distinct signalling events. This may be related to the fact that while *DUSP1*/MKP-1 regulates nuclear MAPK activity, *DUSP10*/MKP-5 can impinge on cytosolic signalling and thus the two MKPs may regulate quite distinct sets of MAPK substrates.

### 2.2 DUSP2

*DUSP2* (also known as PAC-1) was first identified as a mitogen-inducible gene in human T-cells and is most closely related to *DUSP1*/MKP-1 and *DUSP4*/MKP-2, sharing 71% and 68% amino acid identity, respectively [81]. Mainly expressed in hematopoietic tissue, *DUSP2* transcription is induced by activation of the ERK1/2 signalling pathway [81,82]. When expressed in mammalian cells, DUSP2 favours dephosphorylation of ERK1/2 and p38 MAPKs, being less able to inactivate JNK [83]. Its lack of activity against JNK was later suggested to be a result of the relative inability of this MAPK to cause catalytic activation of DUSP2 when compared with ERK2 [84]. In a recent twist, DUSP2 was found to be unique amongst the 10 mammalian MKPs in being able to bind to and dephosphorylate the “atypical” MAPK kinases ERK3 and ERK4 [85]. In both ERK3 and 4 the classical T-X-Y motif in the activation loop is replaced by S-*E*-G, in which the serine residue is the sole phospho-acceptor and DUSP2 efficiently dephosphorylates this residue in cultured cells.

#### 2.2.1 *DUSP2* in innate and adaptive immunity

DUSP2 expression is restricted to thymus, spleen and lymph nodes. However, *DUSP2*^−/−^ mice develop normally and show no abnormalities in the numbers of lymphocytes in blood and bone marrow. Granulocyte numbers and lymphoid tissue development are also normal, indicating that DUSP2 is not required for immune system development [86,87]. However, using the K/B_x_N model of inflammatory arthritis, wild type mice injected with arthritogenic K/BxN serum containing autoantibodies to glucose-6-phosphate isomerase (GPI) developed peripheral inflammatory arthritis within 2 days while *DUSP2*^−/−^ mice were protected. Further analysis showed that *DUSP2*^−/−^ animals had impaired effector responses such as inflammatory mediator production by macrophages and mast cells and decreased mast cell survival [86]. Taken together, these results demonstrate an unexpected role for DUSP2 as a *positive* mediator of inflammation. Puzzlingly, stimulated mast cells and macrophages lacking DUSP2 displayed decreased ERK1/2, and p38 MAPK phosphorylation and increased JNK phosphorylation, which is exactly the opposite of the result predicted by prior biochemical studies [11,84]. No compensatory changes in the expression of other MKPs was observed and the authors invoke pathway crosstalk, postulating that the increase in JNK activity on DUSP2 deletion resulted in suppression of ERK activity.

More recently, Lu et al., have studied the role of *DUSP2* in T cell development and differentiation and found that loss of this phosphatase has a profound effect on the differentiation of naive T cells *in vitro* by favouring Th17 differentiation, while inhibiting the production of into Treg cells [87]. Using the dextran sodium sulfate (DSS)-induced model of intestinal inflammation and colitis, they further show that DUSP2^−/−^ mice exhibit more severe disease when compared to wild type, as evidenced by increased mucosal hyperemia and colonic ulceration. Consistent with the *in vitro* results, this pathology is accompanied by higher levels of Th17 cells in DSS-treated *DUSP2*^−/−^ colon and increased levels of pro-inflammatory cytokines including IL-6, IL-17, TNFα and interleukin-1beta (IL-1β) [87]. Mechanistically, while levels of phospho-ERK and phospho-p38 were higher in untreated DUSP2^−/−^ colon compared to wild type, no differences were seen in DSS-treated colon from the two genotypes. However, higher levels of phospho-STAT3 were consistently seen in mice lacking DUSP2 and the authors hypothesise that this transcription factor is a direct DUSP2 substrate in vivo. However, as JAK/STAT signalling is potently activated in response to IL-6 and this cytokine is overproduced in response to DUSP2 deletion some caution must be attached to this interpretation, particularly as DUSP2 (like DUSP6/MKP-3 see Section 3.1) undergoes catalytic activation by bound ERK2, implying that its full activity as a protein phosphatase is dependent on binding to a MAPK substrate [84].

Taken together, these results demonstrate that DUSP2 plays key roles in both the innate and adaptive immune systems, which have implications for the initiation and progression of pathology in murine models of human inflammatory disease (Table 2). However, at present it is unclear whether or not these relate to the direct activity of this phosphatase in modulating MAPK signalling or may involve other relevant targets. Clearly more work is required to reconcile the in vivo observations with precise molecular mechanism.

#### 2.2.2 DUSP2 in cancer

Thus far DUSP2^−/−^ mice have not been crossed into any of the commonly used murine cancer models and reports of the involvement of DUSP2 in cancer are relatively scant (Table 4). Down-regulation of DUSP2 has been reported in a number of solid tumours, where its expression level was inversely proportional to that of the hypoxia-inducible transcription factor HIF-1α and its loss seemed to mediate increased ERK activation and chemoresistance in cancer cell lines and to contribute to colon cancer “stemness” [88,89]. Given its expression in hematopoietic tissues, there are also a number of studies linking DUSP2 with blood cell cancers. Down-regulation of DUSP2 in acute myeloid leukemia (AML) is associated with constitutive ERK activation [90], while recent data from cancer genome sequencing of Diffuse Large B-cell lymphomas (DLBCL), the major form of non-Hodgkin's lymphoma, reveals that DUSP2 is one of the most frequently mutated genes in this disease [91,92]. The observation that DUSP2 expression is highly inducible upon stimulation of B-cell lymphoma cell lines suggests that mutations in DUSP2 may have the potential to modify MAPK signalling in DLBCL. It will be vital to determine the effects of these mutations on the localisation or activity of DUSP2 in order to explore the possible contribution of this phosphatase to the initiation and/or progression of disease.

### 2.3 *DUSP4*/MKP-2

*DUSP4*/MKP-2 was amongst the very earliest of the MKPs to be characterised and is most closely related to *DUSP1*/MKP-1 [[93], [94], [95]], sharing 58.8% identity at the amino-acid sequence level. Although it is not as widely studied, *DUSP4*/MKP-2 shares many features with its nearest relative including transcriptional regulation in response to growth factors, an ability to dephosphorylate ERK, JNK and p38 MAPKs and regulation of *DUSP4*/MKP-2 protein stability by the phosphorylation of its C-terminus [11,36,96]. The generation of knockout mice has now advanced our knowledge of *DUSP4*/MKP-2 function in a number of areas.

#### 2.3.1 *DUSP4*/MKP-2 in innate and adaptive immunity

The earliest reports using the *DUSP4*^−/−^ mice centred on its possible function as a regulator of innate immunity and inflammation. BMDMs from *DUSP4*^−/−^ mice showed increased levels of both JNK and p38 but not ERK signalling in response to LPS. This correlated with a potentiation of LPS-stimulated induction of the pro-inflammatory cytokines, IL-6, IL-12Beta (IL-12p40), TNFα, and also cyclooxygenase-2 (COX-2) derived prostaglandin E2 (PGE_2_) production [97]. However, IL-10 was suppressed, as was inducible nitric oxide synthase (iNOS) expression while arginase-1 levels were increased. The reciprocal changes in iNOS/arginase-1 levels would tend to suppress nitric oxide (NO) production as arginase-1 competes with iNOS for the same substrate. Following infection with the intracellular parasite *Leishmania Mexicana*, mice lacking *DUSP4*/MKP-2 were found to be more susceptible to infection, with an increased parasite burden and lesion size and this was accompanied by a suppression of Th1 and/or increased Th2 responses. The reason for the increased susceptibility to *Leishmania Mexicana* infection was due to decreased iNOS and increased expression and function of arginase-1 rather than any modulation of cytokine synthesis [97]. Taken together these results suggest that *DUSP4*/MKP-2 does not display simple functional redundancy with respect to its near relative, but instead is protective against *Leishmania Mexicana* infection due to up-regulation of iNOS and suppression of arginase-1 expression, thus promoting NO-mediated parasite death. This mechanism was also found to account for the protective effects of *DUSP4*/MKP-2 against *Leishmania donovani* the causative agent of visceral leishmaniasis [98] and *Toxoplasma gondii*, which causes toxoplasmosis [99].

Differences between *DUSP4*/MKP-2 and *DUSP1*/MKP-1 were further underlined in studies of the response of *DUSP4*^−/−^ mice to experimental LPS-induced sepsis. The first major surprise came with the discovery that, in contrast to mice lacking *DUSP1*/MKP-1, mice lacking *DUSP4*/MKP-2 were more resistant to endotoxic shock and also had *lower* levels of circulating IL-1β, IL-6, and TNFα [100]. Furthermore, LPS-stimulated BMDMs derived from *DUSP4*^−/−^ mice produced significantly less TNFα and IL-10 when compared to wild type cells and this was associated with increased levels of phosphorylated (active) ERK, but decreased levels of phospho-JNK and p38 [100]. It is unclear why there is a discrepancy between these results in LPS-stimulated BMDMs and those obtained by Al-Mutairi et al. [97], but they went on to show that elevated ERK2 signalling led to induction of *DUSP1*/MKP-1 in the *DUSP4*^−/−^ macrophages and that this MKP was responsible for the reduction in JNK and p38 signalling and reduced cytokine production [100]. This supports a model in which ERK-mediated cross talk between MKP-2 and MKP-1 acts to regulate cytokine production in response to LPS, a view supported by the observation that siRNA mediated knockdown of *DUSP1*/MKP-1 increased the production of TNFα by *DUSP4*^−/−^ BMDMs [100].

In the adaptive immune system deletion of *DUSP4*/MKP-2, like deletion of *DUSP1*/MKP-1 [33], does not affect thymocyte maturation and positive selection. Furthermore, no enhanced ERK, JNK, or p38 phosphorylation was observed in either activated or phorbol-12-myristate-13-acetate (PMA)-treated *DUSP4*^−/−^ T cells [101]. However, CD4+, but not CD8+, T cells did show higher rates of proliferation without affecting differentiated Th1 and Th2 T-cell functions in vivo. The proliferative change in CD4+ T cells lacking DUSP4/MKP-2 was associated with increased STAT5 phosphorylation and interleukin 2 receptor alpha (CD25) expression [101]. Subsequent work showed that *DUSP4*/MKP-2 decreases both the transcriptional activity and stability of STAT5 and both *in vitro* and *in vivo* data showed that *DUSP4*/MKP-4 deletion enhanced iTreg and reduced Th17 polarisation while *DUSP4*-deficient mice were somewhat more resistant to the induction of autoimmune encephalitis [102]. Finally, increased *DUSP4*/MKP-2 expression has been implicated in age-dependent defective adaptive immunity. Increased expression of *DUSP4*/MKP-2 in CD4+ memory T cells from older (>65 years) individuals inhibits the ERK and JNK-dependent expression of CD40L and reduces the production of the cytokines interleukin-4 (IL-4) and interleukin-21 (IL-21) by follicular helper cells, thus impairing T cell-dependent B cell responses [103]. These results suggest that specific inhibition of *DUSP4*/MKP-2 activity might form part of a strategy to combat increased morbidity from infections in the elderly.

Taken together these studies clearly implicate *DUSP4*/MKP-2 in the regulation of inmate and adaptive immunity (Table 2). However, despite assumptions that its close relationship with the prototypic MKP *DUSP1*/MKP-1 might indicate overlapping or identical functions this enzyme seems to play a distinct role in regulating immune function. In macrophages there is some debate as to the effects of *DUSP4*/MKP-2 deletion on the activity of specific MAPK isoforms with discordant results in LPS-treated BMDMs [97,100]. In T cells it seems to mediate its effects *via* modulation of STAT5 and, as no perturbation in MAPK signalling was observed in cells lacking *DUSP4*/MKP-2, this has been taken as evidence of a MAPK-independent function for this enzyme [101,102].

#### 2.3.2 *DUSP4*/MKP-2 in cancer

Although less well studied than *DUSP1*/MKP-1, there have nevertheless been numerous reports of either increased or reduced expression of *DUSP4*/MKP-2 in a wide variety of human cancer cell lines and primary tumours including pancreatic, lung, ovarian, breast, liver, thyroid and colon (reviewed in [60,104]). However the majority of these studies have relied on either association and/or correlation with clinical outcome/tumour subtype or ectopic expression of this MKP in cancer cell lines. Thus far the *DUSP4*^−/−^ knockout mice have not been crossed into or utilised in any of the well-characterised murine cancer models and there is no direct evidence of a role for *DUSP4*/MKP-2 in the initiation or progression of tumours. Furthermore, the fact that *DUSP4*/MKP-2 has a proven role in immune regulation indicates that a conditionally targeted allele of *DUSP4*/MKP-2 would be required to avoid the confounding effects of deleting this MKP in immune cells on any cancer phenotypes observed.

Despite this there have been some recent reports that indicate a role for this MKP in human cancers (Table 4). *DUSP4*/MKP-4 was found to be epigenetically silenced in some 75% of >200 cases of diffuse B cell lymphoma (DBCL) and a lack of *DUSP4*/MKP-2 was a negative prognostic factor in three independent cohorts of DBCL patients. Mechanistically, this cancer appears to be dependent on JNK signalling for continued survival and loss of *DUSP4*/MKP-2 contributes to cancer progression by augmenting JNK activity. This is consistent with the results of ectopic expression of *DUSP4*/MKP-2, which ablates JNK activity and induces apoptosis in DBCL cells while dominant negative interference with JNK also restricts survival [105]. There are also indications that *DUSP*2/MKP-4 is implicated in resistance to chemotherapy in both gastric cancer, where resistance to doxorubicin is associated with *DUSP4*/MKP-2 driven epithelial-mesenchymal transition (EMT) [106] and in Her2 positive breast cancer where *DUSP4*/MKP-2 is associated with resistance to the anti-Her2 humanised monoclonal antibody Trastuzumab and siRNA mediated silencing of *DUSP4*/MKP-2 re-sensitised breast cancer cell lines with an amplified Her2 oncogene to this agent [107].

#### 2.3.3 *DUSP4*/MKP-2 in other tissues

*DUSP4*/MKP-2 is expressed at increasing levels during the process of neuronal differentiation in neural progenitors derived from retinoic acid (RA)-treated murine embryonic stem cells (mESCs) and lentiviral *DUSP4*/MKP-2 siRNA knockdown significantly retarded this process. Importantly, this phenotype could be rescued with siRNA-resistant wild type but not a catalytically inactive mutant of *DUSP4*/MKP-2 and loss of *DUSP4*/MKP-2 resulted in increased levels of phospho-ERK, but not JNK or p38 MAPKs, indicating that this is the relevant target. Overall this data indicates that *DUSP4*/MKP-2 plays a role in both the neural commitment of mESCs and neuronal differentiation and may point to a wider role for this MKP in brain function and pathology [108]. More recently direct evidence of a role for *DUSP4*/MKP-2 in the brain has come from a study of hippocampal neuronal excitability, synaptic plasticity and behaviour in *DUSP4*^−/−^ mice. Long-term potentiation (LTP) was found to be impaired in MKP-2^−/−^ mice and the frequency of excitatory postsynaptic current (EPSC) was also increased in both hippocampal slices and hippocampal cultures. Finally, whereas locomotor activity and anxiety-like behaviour were normal in *DUSP4*^−/−^ mice, hippocampal-dependent spatial reference and working memory were both somewhat impaired [109]. Surprisingly, given the established role of ERK signalling in LTP [110] no abnormalities in ERK signalling were observed in either *DUSP4*^−/−^ brain tissue or in primary hippocampal cultures. However, JNK or p38 activation was not studied and the former also play a role in memory formation and synaptic plasticity [111].

### 2.4 *DUSP5*

*DUSP5* was first identified as a growth factor and heat shock-inducible nuclear MKP and is closely related to both *DUSP1*/MKP-1 and *DUSP2*/MKP-4 [[112], [113], [114]]. Despite its early discovery and characterisation as an MKP, little attention was paid to *DUSP5*, presumably on the assumption that it would share many of the properties of its nearest relatives with respect to a broad activity towards ERK, JNK and 38 MAPKs. However, it was later shown that *DUSP5* is unique amongst the four inducible nuclear MKPs in being absolutely specific for ERK1/2 [115]. Furthermore, growth factor-inducible expression of *DUSP5* is mediated by ERK activity making it a classical negative feedback regulator of this signalling pathway [116] and *DUSP5* binds tightly to its substrate and is able to anchor inactive ERK in the cell nucleus [115]. Together, these properties define *DUSP5* as the nuclear counterpart of the inducible cytoplasmic ERK specific phosphatase *DUSP6*/MKP-3 (see Section 3.1).

#### 2.4.1 *DUSP5* in innate and adaptive immunity

An early indication that *DUSP5* might play a role adaptive immunity came with the observation that it was highly induced following IL-2 stimulation of T-cells [117]. This idea was seemingly reinforced by the finding that transgenic expression of *DUSP5* in lymphoid cells arrested thymocyte development at the CD4+/CD8+ (double positive) stage and caused autoimmune symptoms in these animals [118]. However, these results illustrate the limitations of overexpression experiments and probably reflect the function of ERK itself, rather than endogenous *DUSP5* in regulating immune cell development. This has been confirmed by more recent experiments utilising *DUSP5*^−/−^ mice where global deletion had no effect on innate or adaptive immune cell numbers in the bone marrow, spleen or lymph nodes under homeostatic conditions [119]. However, subjecting *DUSP5*^−/−^ mice to acute immune challenges has revealed more subtle phenotypes that are modulated in a *DUSP5*-dependent manner (Table 2).

Thus *DUSP5* has been shown to be highly expressed in eosinophils where it negatively regulates IL-33 mediated survival, *via* the suppression of interleukin-33 (IL-33)-induced ERK-activity and down-regulation of the anti-apoptotic protein B-cell lymphoma-extra large (BCL_x_L). Consequently, *DUSP5*^−/−^ mice challenged by helminth infection display prolonged eosinophil survival, enhanced eosinophil effector functions and were able to clear their parasite burden more efficiently following infection [119]. More recently Kutty et al., while confirming that T cell development is normal in *DUSP5*^−/−^ mice, have shown that in response to acute infection with lymphocytic choriomeningitis virus (LCMV) these animals have decreased numbers of short-lived effector cells (SLECs) and increased proportions of memory precursor effector cells (MPECs). Both cell types are derived from effector CD8+ T cells in response to acute infection with SLEC being highly cytotoxic, cells that readily undergo apoptosis while MPECs retain the ability to proliferate and eventually develop into mature memory T-cells. This defect was intrinsic to T cells as bone marrow chimeric mice in which CD8 + T cells were reconstituted from *DUSP5*^−/−^ donors showed an identical phenotype [120] and this study clearly indicates that *DUSP5* plays an essential role in regulating the survival of SLECs. However, the precise mechanism(s) by which *DUSP5* affects the balance of differentiating and maintaining SLEC and MPEC populations and its dependence on ERK activity are as yet uncertain [120].

#### 2.4.2 *DUSP5* and cancer

The canonical Ras-ERK MAPK signalling pathway is frequently deregulated in human cancers with activating mutations found in upstream components of the pathway including receptor tyrosine kinases (RTKs), Ras GTPases, the MAPK kinase kinase Braf and MAPK kinase (MEK) [121]. The observation that Braf is mutated in 40–60% of malignant melanomas and in tumours of the thyroid, colon and lung underscores the importance of the Ras-ERK pathway in malignant disease, making it an intense focus of anticancer drug discovery [122]. In common with the cytoplasmic ERK-specific phosphatase *DUSP6*/MKP-3 (see Section 3.1.3), elevated *DUSP5* expression is observed in a range of Ras or Braf mutant cancer cells [[123], [124], [125]] where it is presumed to suppress oncogenic ERK activation. *DUSP5* has also been reported to be subject to epigenetic silencing in gastric cancers and this correlated with poorer patient survival [126]. More recently, *DUSP5* down-regulation and promoter hypermethylation has been identified in colorectal tumour samples and cell lines. However, *DUSP5* knockdown in colorectal cancer cell lines displayed limited effects on phospho-ERK levels and did not increase proliferation. Furthermore, a transgenic mouse overexpressing *DUSP5* in the intestinal epithelium displayed no alterations in ERK signalling, intestinal homeostasis or adenoma formation and the authors concluded that *DUSP5* does not regulate intestinal development or tumourigenesis [127]. Although surprising, given the demonstrable effects of *DUSP5* overexpression on ERK activation *in vitro*, these results should be interpreted with a degree of caution as the constitutive transgene used here cannot recapitulate the transcriptional dynamics inherent in feedback control exerted by endogenous *DUSP5*.

**Fig. 4:**
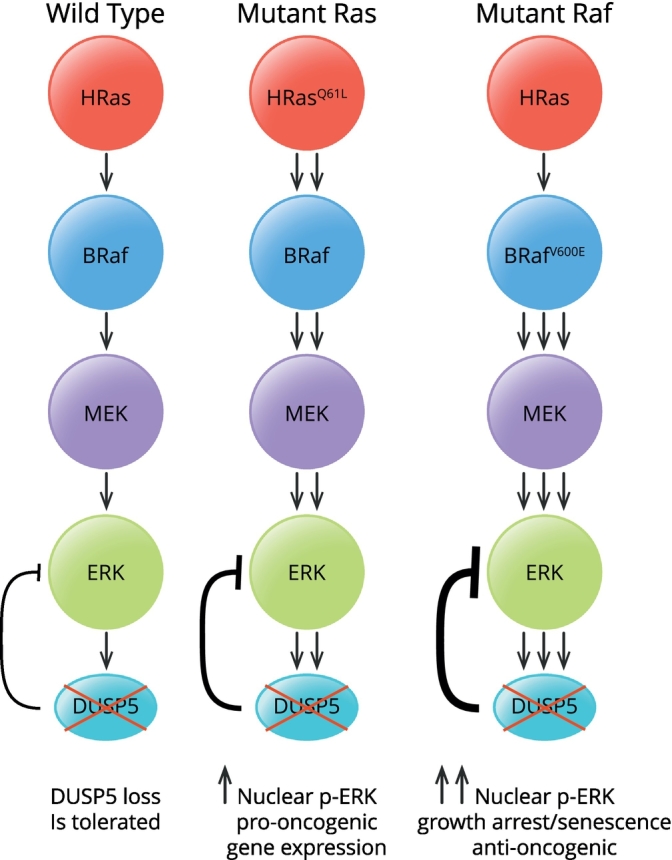
*The effects of DUSP5 deletion on oncogenic signalling through the Ras*-*ERK pathway may depend on the nature of the driving oncogene*. Deletion of *DUSP5* in cells harbouring mutant Hras^Q61L^ causes elevated nuclear ERK activity. This is associated with changes in gene expression and an increased susceptibility to Hras-driven skin carcinogenesis identifying *DUSP5* as a tumour suppressor. In contrast, in MEFs expressing mutant Braf^V600E^ deletion of *DUSP5* causes a much stronger ERK activation that is associated with senescence, growth arrest and suppression of cell transformation indicating that in cancers driven by activated Braf *DUSP5* may be essential for cancer cell survival.

In contrast Rushworth et al. demonstrated that *DUSP5* loss sensitised mice to HRas^Q61L^-driven skin papilloma formation in the well-established DMBA/TPA (7,12-dimethylbenz[*a*]anthracene/12-O-tetra-decanoylphorbol-13-acetate)-inducible skin carcinogenesis model. Furthermore, *in vitro* experiments in *DUSP5*^−/−^ MEFs revealed an essential non-redundant function for this MKP in suppressing nuclear ERK activity following acute pathway stimulation. Loss of *DUSP5*^−/−^ also provoked the upregulation of a cohort of ERK-dependent genes including SerpinB2 in TPA stimulated MEFs [128]. SerpinB2 had previously been identified as a susceptibility gene in this model of skin carcinogenesis [129,130] and concomitant deletion of SerpinB2 reversed the sensitivity of *DUSP5*^−/−^ mice to DMBA/TPA-induced papilloma formation identifying *DUSP5* as a *bona fide* tumour suppressor by virtue of its ability to suppress SerpinB2 expression in this animal model of Ras-induced cancer [128]. More recently, experiments using wild type and *DUSP5*^−/−^ MEFs have demonstrated that *DUSP5* function is dependent on the nature of the oncogenic driver. Thus while loss of this MKP in the context of mutant Ras is compatible with continued cell proliferation, its deletion in cells expressing mutant BRaf^V600E^ causes ERK-dependent cell cycle arrest and senescence and prevents cell transformation by this oncogene *in vitro* [131]. This latter study supports the idea that MKPs might either suppress or promote carcinogenesis depending on the oncogenic and tissue context (Fig. 4, Table 4) and it will be interesting to see the results of *DUSP5* ablation in other, more clinically relevant, murine models of Ras- and Braf-driven cancer.

#### 2.4.3 *DUSP5* in other tissues

*DUSP5* is implicated in cardiovascular development, where it is expressed in angioblasts and mature vasculature in zebrafish and *DUSP5* knockdown increased the etsrp^+^ (ets1-related protein) angioblast population during early embryonic development. *DUSP5* overexpression also antagonised the function of a serine threonine kinase, Snrk-1, which promotes angioblast development [132]. *DUSP5* has also been shown to act as a regulator of cardiac fibroblast proliferation and cardiac hypertrophy. Ferguson et al., demonstrated that the anti-hypertrophic activity of class I histone deacetylase (HDAC) inhibitors is mediated by their ability to increase *DUSP5* gene expression, thus inhibiting both ERK activity and cardiac myocyte proliferation. Ectopic *DUSP5* expression phenocopied the effects of HDAC inhibition while *DUSP5* knockdown rescued endogenous phospho-ERK levels following HDAC inhibition [133]. A subsequent study has revealed that HDAC3 specific inhibitors also induce *DUSP5* expression in mouse models of diabetic cardiomyopathy and that this is associated with reduced hypertrophy and fibrosis [134]. *DUSP5* expression is also suppressed by another epigenetic regulator Methyl-CpG-binding protein 2 (MeCP2) and high MeCP2 expression was associated with cardiac fibrosis and MeCP2 knockdown in cardiac fibroblasts, increased *DUSP5* expression and reduced both ERK activity and proliferation [135]. Finally, in adult rats *DUSP5* deletion has been shown to modulate the myogenic response of cerebral arteries and autoregulation of cerebral blood flow, indicating that *DUSP5* also plays a physiological role in vascular reactivity [136].

## 3 The cytoplasmic ERK-specific MKPs

### 3.1 *DUSP6*/MKP-3

First characterised as an inducible cytoplasmic MKP, which is prototypic of a subfamily of 3 highly related enzymes, *DUSP6*/MKP-3 was subsequently found to display absolute substrate specificity for ERK1 and ERK2 having no significant activity towards JNK, p38 or ERK5 [12,[137], [138], [139], [140]]. This selectivity is mediated by high affinity binding of ERK to the KIM within the amino-terminal domain of *DUSP6*/MKP-3 and underpinned by catalytic activation of *DUSP6*/MKP-3 involving a conformational change within the PTPase domain that repositions key active site residues and greatly increases enzyme activity [141,142]. The cytoplasmic localisation of *DUSP6*/MKP-3 is mediated by a leucine-rich nuclear export signal (NES) within the amino terminal domain and the tight binding of ERK to the KIM also indicates a role for this MKP as a cytoplasmic anchor for inactive ERK [143]. Studies of the pattern of *DUSP6*/MKP-3 expression during early mouse development established a link with sites of fibroblast growth factor (FGF) signalling [144] and subsequent work in the chicken embryo, *DUSP6*^−/−^ mice and cultured cells established that *DUSP6*/MKP-3 is transcriptionally induced in response to FGF-mediated ERK signalling and acts as a classical negative feedback regulator of ERK activity during early development [[145], [146], [147], [148], [149]].

#### 3.1.1 *DUSP6*/MKP-3 in immunity and inflammation

Relatively few studies have addressed a role for *DUSP6*/MKP-3 in immune regulation. *DUSP6*^−/−^ mice are reported to be indistinguishable from their wild type littermates in terms of the total number of cells and proportions of CD4^+^, CD8^+^, and CD4/CD8 double-positive cells in spleen, mesenchymal lymph nodes (MLN) and thymus [150]. However, T cell receptor (TCR) stimulation of DUSP6^*−/−*^ CD4^+^ T cells resulted in higher levels of phospho-ERK1/2, but not of JNK or p38 and anti-CD3/28 stimulated CD4^+^ T cells harvested from spleen and MLN produced higher amounts of IFN-γ and lower amounts of IL-17A when compared to wild type controls. Activated DUSP6^−/−^ CD4^+^ T cells also displayed increased proliferation *in vitro*, but this was accompanied by increased levels of activation-induced cell death (AICD), perhaps explaining why lymphoid cellularity remains unchanged. In *DUSP6*^−/−^ CD8^+^ T cells the expression of CD107a or lysosomal associated membrane protein 1; (LAMP-1) a marker of lymphocyte degranulation, was reduced suggesting that *DUSP6*/MKP-3 may also regulate the cytotoxic activity of CD8^+^ T cells.

The changes in cytokine production by CD4+ T cells after *DUSP6*/MKP-3 deletion are suggestive of a role in T cell polarisation. When subjected to Th1 polarizing conditions a larger number of *DUSP6*^−/−^ CD4^+^ T cells produced IFN-γ, while under Th17 polarizing, conditions *DUSP6*^−/−^ CD4^+^ T cells gave rise to fewer IL-17A producing cells. Taken together these results indicate that *DUSP6*/MKP-3 regulates the polarisation of CD4^+^ T cell subsets by inhibiting Th1 differentiation and favouring Th17 differentiation. Furthermore, to assess the function of *DUSP6*^−/−^ regulatory T cells (Treg) these were isolated and co-cultured with naïve CD4^+^ T cells from WT mice and stimulated with anti-CD3/CD28 for 72 h before assessing CD4+ T cell proliferation. *DUSP6*−/− Treg cells consistently showed a lower capacity to inhibit the proliferation of naïve CD4^+^ T cells indicating that *DUSP6*/MKP-3 is required for suppressive Treg function.

Finally, to assess *DUSP6*/MKP-3 function *in vivo DUSP6*^−/−^ mice were crossed with IL-10^−/−^ mice and assessed for the development of intestinal colitis. The double knockout (DKO) mice consistently developed severe inflammation with epithelial crypt hyperplasia, loss of goblet cells, and immune cell infiltration into colonic connective tissue while DKO colonic explants produced increased levels of IFN-γ and TNFα, but lower levels of IL-17A when compared to IL-10^−/−^ tissues. Satisfyingly, administration of PD0325901, a specific MEK inhibitor, both ameliorated and reversed the inflammatory phenotype seen in the DKO mice demonstrating that this is a direct result of increased levels of ERK1/2 signalling in the absence of *DUSP6*/MKP-3 [150].

In a recent study of the role of *DUSP6* in endothelial cell inflammation Hsu et al. found that tail vein injection of TNFα caused elevated expression of Intercellular Adhesion Molecule 1 (ICAM1) in wild type but not *DUSP6*^−/−^ animals, suggesting that *DUSP6* would modulate neutrophil recruitment and transendothelial migration at sites of acute inflammation. In agreement with this hypothesis, *DUSP6*^−/−^ animals injected intraperitoneally with either TNFα or LPS showed lower levels of pulmonary neutrophil infiltration and lung injury and adoptive transfer of wild type neutrophils into *DUSP6*^−/−^ mice revealed that the defect was intrinsic to endothelial cells [151]. Surprisingly, *in vitro* experiments in primary human umbilical vein endothelial cells (HUVECs) revealed that the role of *DUSP6*/MKP-3 in regulating TNFα-induced expression of ICAM1 involved activation of canonical nuclear factor (NF)-κB but was not dependent on its ability to dephosphorylate ERK MAPK [151].

Taken together, these studies indicate that *DUSP6*/MKP-3 may play complex and tissue specific roles in immune cell function and inflammatory processes (Table 2) and further work will be required to delineate the precise nature of the signalling events involved and the tissue specificity of *DUSP6*/MKP-3 functions.

#### 3.1.2 *DUSP6*/MKP-3 in metabolic homeostasis

The first indication that *DUSP6*/MKP-3 might be involved in metabolic control was the finding that its expression was able to prevent the suppression of phosphoenolpyruvate carboxylase (PEPCK) gene expression by insulin. *DUSP6*/MKP-3 was also expressed in insulin-responsive tissues and expression levels were markedly elevated in the livers of insulin-resistant genetically obese (db/db) mice [152]. Subsequent work showed that expression of *DUSP6*/MKP-3 is also increased in the livers of HFD-induced obese mice and that adenovirus-mediated *DUSP6*/MKP-3 expression in lean mice promoted gluconeogenesis and increased levels of fasting blood glucose. In contrast, shRNA knockdown of *DUSP6*/MKP-3 in both lean and obese mice resulted in decreased fasting blood glucose levels [153]. Mechanistically the transcriptional upregulation of gluconeogenic genes such as PEPCK that underpinned these effects was mediated by the dephosphorylation and nuclear translocation of Forkhead box protein O1 (FOXO1). Surprisingly, the effects of *DUSP6*/MKP-3 on FOXO1 were postulated to be direct, *via* protein-protein interaction and dephosphorylation of this transcription factor [154]. However, this finding is extremely difficult to reconcile with the known biochemical properties of *DUSP6*/MKP-3 and in particular the requirement for ERK2 binding to achieve catalytic activation of this phosphatase [141].

Experiments utilising *DUSP6*^−/−^ mice to study metabolism have now been performed with the finding that mice lacking *DUSP6*/MKP-3 are somewhat protected from diet-induced obesity (Table 3). However, quite different conclusions were reached regarding the underlying mechanism. Feng et al. reported that *DUSP6*^−/−^ mice are protected against both HFD-induced weight gain and hepatosteatosis and that these effects were accompanied by reduced liver Triglyceride (TG) levels and adiposity. *DUSP6*^−/−^ mice also exhibited increased energy expenditure, enhanced peripheral glucose disposal, and improved systemic insulin sensitivity [155]. Phosphoproteomic analyses in cultured Hepa1–6 cells +/− siRNAs targeting *DUSP6*/MKP-3 and comparing liver lysates from *DUSP6*^−/−^ and *DUSP6*^+/+^ mice revealed significant increases in the phosphorylation of HDAC1 and 2. Pharmacological inhibition or combined knockdown of these enzymes in primary hepatocytes from *DUSP6*^−/−^ mice was able to reverse the protective effects of *DUSP6*/MKP-3 deletion by raising the expression levels of several lipogenic genes, indicating that these enzymes may be the relevant *in vivo* targets [155].

Ruan et al. also reported protection against HFD-induced weight gain in *DUSP6*^−/−^ mice together with improved glucose tolerance, increased insulin sensitivity and protection against hepatosteatosis [156]. However, faecal transplantation from HFD-fed *DUSP6*^−/−^ mice into germ-free animals phenocopied this resistance and following studies of *DUSP6*/MKP-3-dependant changes in gut microbiota, intestinal barrier function and the gut transcriptome they concluded that *DUSP6*/MKP-3 loss protects the intestinal epithelial barrier from HFD-induced interruption and subsequent remodelling of the gut flora to maintain a lean-associated microflora. They conclude that *DUSP6*/MKP-3 regulates homeostasis between the gut epithelium, mucosal immunity and microbiota [156]. In contrast, a recent study observed comparable body weight, fat and lean mass in *DUSP6*^−/−^ and *DUSP6*^+/+^ mice after 26 weeks on a HFD. However, glucose tolerance was somewhat abnormal in both lean and obese *DUSP6*^−/−^ mice when compared to controls [157]. At present it is unclear why there is a discrepancy between the latter study and the previous two, but the finding that variations in the gut microbiota can have profound consequences for sensitivity to HFD coupled with differences in the genetic backgrounds used in these studies (pure C57Bl/6 J *vs*. mixed 129 x C57Bl/6 J) may account for this. As was the case for studies of *DUSP1*/MKP-1, the use of an unconditional (whole body) knockout of *DUSP6*/MKP-3 also makes the interpretation of these studies more difficult and future experiments using conditional ablation of *DUSP6*/MKP-3, will be required to address complexity and tissue specific interplay in the regulation of metabolic homeostasis by this phosphatase.

#### 3.1.3 *DUSP6*/MKP-3 and cancer

Given the direct involvement of Ras/ERK signalling in human cancer a possible role for *DUSP6*/MKP-3 has been explored in some depth (Table 4). As was the case for the inducible nuclear ERK-specific phosphatase *DUSP5*, increased *DUSP6*/MKP-3 expression has been observed in primary tumours and cancer cell lines, which harbor mutations in either Ras or Braf, where its role as a negative feedback regulator of ERK activity has led to the suggestion that it may act as a tumour suppressor [125,158,159]. Thus *DUSP6* expression is initially elevated and then epigenetically silenced during the progression of mutant Kras-driven pancreatic ductal adenocarcinoma, with the lowest levels detected in the most invasive and poorly differentiated tumours [[160], [161], [162]]. In a similar vein, loss of *DUSP6*/MKP-3 expression in mutant Kras-driven lung tumours is associated with increased disease severity and histological grade and loss of heterozygosity at the *DUSP6* locus occurs in almost 20% of patients [163]. However, as is the case for many MKPs much of the mechanistic data obtained so far involves the reversal of cancer-associated phenotypes by ectopic expression of *DUSP6*/MKP-3 in cancer cell lines [161,163] and must therefore be treated with a degree of caution. No direct evidence of a tumour suppressor function for this MKP has yet been obtained by crossing *DUSP6*^−/−^ mice into established murine cancer models. In contrast, two recent reports contain evidence that *DUSP6*/MKP-3 may actually be an oncogenic driver in certain human cancers.

Firstly, Shojaee et al., found that the acute oncogenic activation of BCR-Abl and Nras^G12D^ in human pre-B cells was invariably lethal. However, any surviving cells were transformed and displayed increased expression of negative regulators of ERK signalling including DUSP6/MKP-3. Furthermore, this upregulation of *DUSP6*/MKP-3 was also seen in pre-B ALL cells, where it was driven by both Abl and ERK activity and high DUSP6/MKP-3 mRNA levels in patients with Philadelphia chromosome positive (Ph^+^) (BRC-Abl-driven) ALL was associated with shorter survival [164]. To gain mechanistic insight, Shojaee et al. transformed bone marrow-derived B cell lineage progenitor cells from DUSP6^*−/−*^ mice and wild-type controls with BCR-Abl1. Interestingly, the survival of the DUSP6^−/−^ cells was significantly reduced and conditional expression of Nras^G12D^ in pre-B cells was only able to transform wild type but not DUSP6^−/−^ cells. siRNA-mediated knockdown of *DUSP6*/MKP-3 in human pre-B ALL cells also reduced survival. All of these observations strongly indicate that pre-B ALL cells are dependent on DUSP6/MKP-3-mediated negative feedback control of ERK signalling for continued survival and growth [164]. In support of this conclusion, BCI, a compound identified as an allosteric inhibitor of *DUSP6*/MKP-3, caused a rapid increase in ERK activity in patient-derived Ph^+^ ALL cells. Mouse xenograft experiments using Ph^+^ ALL cells derived from patients after relapse during ongoing therapy with the Abl inhibitor Imatinib (Gleevec) showed these to be resistant to tyrosine kinase inhibition, but sensitive to treatment with BCI, indicating that this drug might be used to treat TKI-resistant Ph^+^ ALL [164]. While these results are very provocative, they must be treated with a degree of caution in that BCI, as mentioned previously in the context of its use as an inhibitor of *DUSP1*/MKP-1, is known to be relatively non-specific and displays considerable off-target toxicity [71].

In a recent genetic screen Wittig-Blaich et al., identified *DUSP6*/MKP-3 amongst a set of genes with growth suppressive properties consistent with tumour suppressor function. However, specifically in the context of mutant Braf–driven melanoma they found that siRNA-mediated knockdown of *DUSP6*/MKP-3 caused apoptosis. They speculate that *DUSP6*/MKP-3 might be required to prevent Braf^V600E^ hyperactivation from triggering cell death *via* ERK1/2 downstream substrates and suggest that it might represent a synthetic lethal drug target in this subset of melanoma patients [165]. The idea that MKPs can intervene to prevent the engagement of tumour suppressive pathways has been suggested previously, mainly in the context of ERK-dependent oncogene-induced senescence (OIS) [166] and, as discussed previously, there is some support for differential outcomes in terms of cell proliferation and senescence after deletion of the inducible nuclear ERK-specific MKP *DUSP5* in cells expressing either activated Ras or Braf [131]. In support of the idea that *DUSP6*/MKP-3 may be pro-oncogenic in certain cancer types DUSP6/MKP-3 is upregulated in human glioblastoma cell lines and mouse xenograft experiments showed that tumours arising from glioblastoma cells expressing DUSP6/MKP-3 grew significantly faster than non-expressing controls [167]. The overexpression of DUSP6/MKP-3 in papillary thyroid carcinoma (PTC) cell lines is also associated with increased cell migration and invasion [168].

Finally, although *DUSP1*/MKP-1 has been widely studied as a modulator of drug responses in cancer chemotherapy (see Section 2.1.3) less attention has been paid to the ERK-specific MKPs. However, Phuchareon et al. recently identified the down-regulation of *DUSP6*/MKP-3 as a contributing factor to the reactivation of Ras-ERK signalling and drug resistance in epidermal growth factor receptor (EGFR) mutant lung cancer cell lines exposed to the TKI's gefitinib (Iressa) and erlotinib (Tarceva) [169]. Resistance was mediated by increased ERK-dependent phosphorylation and degradation of the extra-long isoform of the pro-apoptotic B-cell lymphoma-2 (Bcl-2) family protein Bim (BimEL) thus promoting cancer cell survival. Interestingly, *DUSP6*/MKP-3 down-regulation has also been implicated in mediating reactivation of Ras-ERK signalling and drug resistance in lung cancer cells harbouring the echinoderm microtubule-associated protein-like 4 -anaplastic lymphoma kinase (ELM4-ALK) fusion protein exposed to the TKI crizotinib (Xalkori) [170]. As one of these two driver mutations are present in almost 20% of non-small cell lung cancers (NSCLC), targeting ERK signalling in combination with the use of TKI's may be a viable strategy to forestall or prevent TKI resistance and loss of *DUSP6*/MKP-3 is worthy of wider study as a possible ERK-mediated drug resistance mechanism in other tumour types driven by abnormal tyrosine kinase activity.

In conclusion it is perhaps no surprise that *DUSP6*/MKP-3 is implicated in the regulation of oncogenic signalling through the Ras/ERK pathway and recent work indicates that the effects of altered expression levels may depend on both the oncogenic background the tissue(s) involved (Table 4). Further studies will be required, preferably using conditional deletion of this MKP in murine models to dissect out its precise role in carcinogenesis, particularly given its emerging role in immune regulation and inflammatory processes, both of which may have a bearing on tumour initiation and development.

### 3.2 *DUSP7*/MKP-X

*DUSP7*/MKP-X most closely related to *DUSP6*/MKP-3 and shares many properties, including cytoplasmic localisation, a high degree of substrate specificity for the ERK1/2 MAPKs and substrate-induced catalytic activation [12,137,171] but, in contrast to *DUSP6*/MKP-3, almost nothing is known about its physiological function(s) or association with human disease. However, *DUSP7*/MKP-X was identified in an siRNA based phenotypic screen for genes involved in meiotic progression in mouse oocytes [172] and more recent work has shown that *DUSP7*-depleted oocytes either fail or are significantly delayed in resuming meiosis and that cyclin-dependent kinase-1/cyclin B (Cdk1/CycB) activity drops below the critical level required to reinitiate meiosis. Once in meiosis *DUSP7*/MKP-X depleted oocytes also had severe chromosome alignment defects and progressed into anaphase prematurely [173]. These effects were judged to be secondary to a failure to dephosphorylate and inhibit protein kinase C isoforms, a prerequisite for the timely activation of Cdk1/CycB and are likely to be significant as both male and female mice lacking *DUSP7*/MKP-X have been reported as viable but infertile (http://www.mousephenotype.org/data/genes/MGI:2387100). As failure to resume oocyte meiosis is a contributing factor to female infertility in humans it will be important to establish whether this signalling pathway is conserved.

### 3.3 *DUSP9*/MKP-4

MKP-4, the third member of this group of cytoplasmic phosphatases, is encoded by the X-linked *DUSP9* gene and has properties in common with *DUSP6*/MKP-3 and *DUSP7*/MKP-X, although its substrate specificity is somewhat more relaxed with respect to binding and inactivation of JNK and p38 MAPKs [141,174,175]. *DUSP9*/MKP-4 is also somewhat unusual in that it is not transcriptionally regulated in response to either growth factor simulation or stress, but instead seems to be constitutively expressed and regulated *via* phosphorylation of a conserved serine residue adjacent to the KIM, which abrogates substrate binding [176]. Unconditional deletion of *DUSP9* results in embryonic lethality due to placental insufficiency, but tetraploid rescue experiments demonstrated that it is otherwise dispensable for normal embryonic development [177]. Information about possible physiological roles for *DUSP9* and links to human disease is relatively scant. Selective expression of *DUSP9* expression in Plasmacytoid dendritic cells (pDCs), but not conventional dendritic cells (cDCs), suggested a possible role in determining this phenotype. However, conditional deletion of *DUSP9* in pDCs did not increase ERK activation after TLR9 stimulation and only weakly affected IFN-β and IL-12Beta (IL-12p40) production by these cells indicating that this MKP is not essential for the high level production of IFN-β, which is characteristic of pDCs [178]. However, recent work now indicates a link between *DUSP9*/MKP-4 and metabolic homeostasis (Table 3).

In obese and insulin resistant mouse models *DUSP9*/MKP-4, protein levels are reported to be elevated in insulin responsive tissues and expression of *DUSP9*/MKP-4 caused increased expression of PEPCK. Overexpression of *DUSP9*/MKP-4 in adipocytes also blocked insulin-stimulated glucose uptake, again suggesting that this enzyme antagonises the effects of insulin in responsive cells and tissues [179]. However, in a stress-induced *in vitro* model of insulin resistance and following adenoviral-mediated overexpression of *DUSP9*/MKP-4 in the livers of genetically obese (ob/ob) mice Emanuelli et al. reported that *DUSP9*/MKP-4 expression improved glucose intolerance, decreased the expression of gluconeogenic genes and reduced hepatic steatosis [180]. Despite these apparently contradictory results, a recent study in which conditional liver-specific deletion of *DUSP9*/MKP-4 was used to study the response to a high fat diet has demonstrated that loss of *DUSP9*/MKP-4 in the liver sensitises animals to hepatic steatosis and inflammatory responses and that *DUSP9* deficiency aggravated high fat, high cholesterol (HFHC)-induced liver fibrosis [181]. In this context it will be interesting to compare the effects of whole body *DUSP9* KO and also loss of this MKP in other insulin responsive tissues as studies of *DUSP1*/MKP-1 function in metabolic homeostasis have revealed that the liver phenotype may not be dominant over the effects of loss of function in other tissues [50,56,57]. Finally, a genetic variant mapping near to the *DUSP*9 gene locus has repeatedly been detected in genome-wide association studies (GWAS) as a risk factor for the development of Type-2 diabetes across different ethnicities in human populations, further reinforcing a link between *DUSP9*/MKP-4 and metabolic control [[182], [183], [184]]. The availability of a conditionally targeted allele for *DUSP9* should greatly accelerate future work on the possible role of this MKP, both in metabolic disease and in other pathologies.

## 4 The JNK and p38 specific MKPs

### 4.1 DUSP8

Along with *DUSP*7/MKP-X, *DUSP8* is probably the least studied of the 10 dual-specificity MKPs and there is virtually nothing known about its physiological function. Since its identification as a gene encoding an MKP with a translated complex trinucleotide repeat within its coding region and characterisation of its specificity for the inactivation of JNK and p38 MAPKs [138] fewer than 20 papers have been published on *DUSP8* and although targeted mouse ES cells have been generated by the international mouse phenotyping consortium (IMPC), these have not yet been exploited to produce a mouse model.

### 4.2 *DUSP10*/MKP-5

*DUSP10*/MKP-5 was first characterised as a widely expressed JNK and p38 specific MKP, which when expressed in mammalian cells is found in both the cytoplasm and nucleus [185,186]. One unique feature of *DUSP10*/MKP-5 is that it carries an amino-terminal extension of unknown function, but which may carry signals that specify its subcellular localisation [185].

#### 4.2.1 *DUSP10*/MKP-5 in immunity and inflammation

Along with *DUSP1*/MKP-1, *DUSP10*/MKP-5 was one of the first MKPs found to regulate both innate and adaptive immune function. While development of the myeloid and lymphoid lineages was normal in mice lacking *DUSP10*/MKP-5, the *DUSP10*/MKP-5 gene is inducible at the transcriptional level in response to TLR signalling and peritoneal macrophages lacking *DUSP10*/MKP-5 showed increased production of the pro-inflammatory cytokines IL-6 and TNFα. Consistent with this, LPS injected *DUSP10*^−/−^ mice had higher serum levels of TNFα when compared to wild type controls. Mechanistically, JNK appeared to be the relevant *DUSP10*/MKP-5 substrate as elevated levels of phospho-JNK were observed in both macrophages and T cells derived from *DUSP10*^−/−^ mice [187]. However, subsequent experiments have clearly shown that *DUSP10*/MKP5 does regulate p38 MAPK activity in both monocyte/macrophages [188] and neutrophils [189] indicating that both stress activated MAPK pathways are subject to negative regulation by *DUSP10*/MKP-5.

As discussed previously in relation to the function of *DUSP1*/MKP-1 [35], an important function of the innate immune system is mediated *via* professional antigen presenting cells (APC), which activate antigen specific T cells, thus bridging the innate and adaptive immune systems [34]. LPS treated APCs isolated from *DUSP10*^−/−^ mice exhibited enhanced priming of ovalbumin-transgenic OT-I (CD4+) and OT-II (CD8+) T cells as assessed by increased levels of IL-2 production and T cell proliferation when compared to wild type APCs. Taken together these results indicate that *DUSP10*/MKP-5 plays a non-redundant role as a negative regulator of the innate immune response [187].

In terms of T cell differentiation and function *DUSP10*^−/−^ Th1 and Th2 cells showed increased JNK activation, but this was lost rapidly on anti-CD3 re-stimulation, indicating that *DUSP10*/MKP-5 is not the sole arbiter of JNK activity in these cells. Activation of CD4+ T cells from *DUSP10*^−/−^ mice with anti-CD3 (with or without anti-CD28 co-stimulation) resulted in reduced proliferation when compared to wild type, indicating that *DUSP10*/MKP-5 is required for T cell expansion. However, IFN-γ production by activated Th1 and IL-4 production by activated Th2 cells lacking *DUSP10*/MKP-5 were increased, while activated effector CD8+ T cells from *DUSP10*^−/−^ mice produced more IFN-γ and TNFα than wild type cells [187]. In concordance with these results, immunisation of *DUSP10*^−/−^ mice with keyhole limpet haemocyanin (KLH) and re-stimulation *ex vivo*, resulted in reduced antigen-driven proliferation of splenic T cells but increased levels of IFN-γ and IL-4 production confirming the reciprocal role of *DUSP10*/MKP-5 in regulating of T-cell clonal expansion and effector T-cell cytokine expression [187]. Finally, the role of *DUSP10*/MKP-5 in susceptibility to infection with LCMV and to MOG-induced EAE was tested. While *DUSP10*^−/−^ mice showed little difference in their initial primary T cell response to infection or in viral clearance, re-challenge caused immune–mediated death, probably as a result of the markedly elevated levels of serum TNFα produced by CD4+ and CD8+ T cells. In the EAE model *DUSP10*^−/−^ mice exhibited a reduced number of CD4 T cells in the brain, which correlated with reduced incidence and severity of disease, indicating that this MKP plays a positive role in the generation and/or expansion of autoreactive T cells in this autoimmune disease model [187].

More recent work has explored the role of this MKP in regulating host responses to inflammatory stimuli using a mouse model of local Shwartzman reaction (LSR). LSR is a delayed vascular injury produced by sequential subcutaneous injection of LPS and TNFα and *DUSP10*^−/−^ mice were found to be much more susceptible to this form of injury. Mechanistically this was the result of increased p38 MAPK activation in neutrophils and the production of greatly increased levels of superoxide anion by the NADPH oxidase complex, thus revealing an essential role for *DUSP10*/MKP-5 as a negative regulator of p38-mediated neutrophil reactive oxygen intermediate (ROI) production, [189]. Interestingly, p38-mediated cytokine and ROS production by macrophages also accounts for the increased susceptibility of *DUSP10*^−/−^ mice to endotoxin-induced acute lung injury following intratracheal injection of LPS, again reinforcing the role of this MKP in protection against inflammatory tissue injury [190].

Collectively these studies reveal that *DUSP10*/MKP-5 like *DUSP1*/MKP-1 plays an important role in regulating both innate and adaptive immune responses (Table 2) and reveals significant complexity and tissue specificity in its interactions with MAP kinase signalling. This is illustrated by the observation that while the defect in T cell expansion seen on loss of *DUSP10*/MKP-5 is responsible for protection against EAE, this deficit does not result in a reduction in the numbers of LCMV-reactive T cells following viral infection, probably because of the compensatory effects of *DUSP10*/MKP-5 loss in stimulating APC function.

#### 4.2.2 *DUSP10*/MKP-5 function in other tissues

Shi et al. have reported a function for *DUSP10*/MKP-5 in regulating muscle stem cell function and muscle regeneration. *DUSP10*^−/−^ mice had increased levels of p38 and JNK activation, muscle mass and muscle fibre size when compared to wild type animals. Furthermore, in response to muscle injury following injection of cardiotoxin, they display an enhanced regenerative response associated with early upregulation of JNK and later of p38 activity. Interestingly, when crossed into the *mdx* (dystrophin null) mouse model of Duchenne's muscular dystrophy, the double knockout animals showed an amelioration of disease manifested by improved skeletal muscle morphology, a reduced number of degenerating muscle fibres and improved contractile function. Mechanistically, this was underpinned by increased muscle stem cell proliferation and differentiation, which were regulated by increased JNK-mediated expression of cyclin D and p38 mediated myogenesis respectively. Finally, despite its function as an immune regulator, these effects of *DUSP10*/MKP-5 loss appeared to be completely independent of alterations in immune cell infiltration into damaged muscle [79].

Interestingly, the effects of *DUSP10*/MKP-5 deletion on myogenesis appear to be due to two main effects. Firstly, deletion of *DUSP10*/MKP-5 increases MAPK-dependent phosphorylation of guanine nucleotide exchange factor for the Ras-related protein Rab-3A Rab3A (GRAB) at Serine 169, a site required for secretion of the promyogenic cytokine IL-6 [191]. Secondly, in the absence of *DUSP10*/MKP-5 increased JNK and p38 mediated phosphorylation and activation of STAT3, increases the expression of the anti-apoptotic protein B-cell lymphoma 2 (Bcl2) thus preventing apoptosis during regenerative myogenesis and also leads to improved antioxidant defence capacity due to a sustained increase in catalase expression that protects mitochondrial function [192].

Finally, a recent study has used *DUSP10*^−/−^ mice to address the possible function of this MKP in modulating the development of DSS-induced intestinal inflammation and colitis associated cancer (CAC) (Table 4). Surprisingly, given the previous findings that mice lacking DUSP10/MKP-5 were more sensitive to inflammatory tissue damage in skin and lung [189,190], DSS treated DUSP10^−/−^ mice exhibited lower levels of intestinal inflammation, better intestinal crypt architecture and lower levels of pro-inflammatory cytokine/chemokine expression than wild type animals. This protection was secondary to improved intestinal epithelial cell (IEC) barrier function, which serves to separate luminal contents from the mucosal immune system, as evidenced by reduced IEC leakage of fluorescein isothiocyanate (FITC)-dextran [193]. Mechanistically, this was due to increased ERK–mediated expression of Kruppel like factor-5 (KLF5) a transcription factor which up-regulates cyclinB expression and promotes IEC proliferation during intestinal regeneration and wound healing. In accordance with this increased numbers of proliferating Ki67-positive cells were observed in the intestinal crypts of DSS-treated *DUSP10*^−/−^ colon compared with wild type tissue [193]. Again these results suggest possible tissue specific variation in *DUSP10*/MKP-5 activity towards MAPKs as ERK but not JNK or p38 activity was affected by *DUSP10*/MKP-5 loss. Finally, although protective against DSS-induced inflammation, the combined treatment of *DUSP10*^−/−^ mice with the mutagen azoxymethane (AOM) and DSS resulted in an increased incidence of adenomatous polyps of larger size, which stained positive for Ki67 and β-catenin. Overall, this strongly suggests that the IEC and the subsequent tumours were more proliferative in the absence of *DUSP10*/MKP-5 indicative of a tumour suppressor function for this MKP, an idea supported by the observation that higher levels of *DUSP10*/MKP-5 expression correlated with better survival amongst patients with colorectal cancer [193].

### 4.3 *DUSP16*/MKP-7

*DUSP16*/MKP-7 was the last of the 10 dual-specificity MKPs to be identified and was characterised as a JNK and p38-specific MKP with a possible function as a regulator of JNK activity in macrophages [[194], [195], [196]]. Although relatively little is known about this phosphatase, three recent studies using knockout mice have begun to shed some light on its possible physiological role(s). Firstly, using a gene trap null mutation Niedzielska et al. reported that loss of *DUSP16*/MKP-7 caused perinatal lethality [197]. Observing that *DUSP16*/MKP-7 was inducible in macrophages in response to TLR agonists, they used fetal liver cells from the null mice to reconstitute the lymphoid and myeloid lineages in lethally irradiated syngeneic CD45.1+ animals. They found that T and B cell populations were present in the normal numbers, that >95% of resident macrophages were derived from the *DUSP16*/MKP-7 null donor cells and the mice had normal numbers of granulocytes and plasmacytoid dentritic cells, indicating that *DUSP16*/MKP-7 is not essential for homeostasis of the immune system under steady state conditions. However, there was a deficit in numbers of splenic CD11c+/CD11b + myeloid dendritic cells secondary to impaired granulocyte-macrophage colony-stimulating factor (GM-CSF)-driven proliferation of bone marrow progenitors [197]. Subjecting reconstituted mice to LPS challenge revealed no undue sensitivity to sepsis, but did reveal JNK-dependent overproduction of IL-12Beta (IL-12p40) by macrophages in response to LPS [197]. Overall these results reveal a dual function for *DUSP16*/MKP-7 in the innate immune system involving selective control of differentiation and cytokine production (Table 2), but further work is required to map out the physiological consequences of this regulation.

Zhang *et al* also reported that loss of *DUSP16*/MKP-7 was lethal and used reconstitution experiments to study the role of this MKP in adaptive immunity (Table 2) [198]. In agreement with Niedzielska et al. [178], they found that T cell development and numbers were normal, but that CD4+ T cells lacking *DUSP16*/MKP-7 were hyper-responsive to activation, produced much more IL-2 and had higher rates of proliferation when compared to wild type cells. To study T cell differentiation and function they cultured naive *DUSP16*^−/−^ CD4^+^ T cells under Th1, Th2, or Th17 conditions *in vitro* and found that while functional Th1 and Th2 cells were produced normally, *DUSP16*^−/−^ Th17 cell populations produced less IL-17A and IL-17F, and contained nearly 50% less IL-17A–producing cells compared with WT cells [198]. Interestingly, U0126 a specific MEK inhibitor, efficiently reversed the deficit in IL-17A producing Th17 cells *in vitro*, indicating that regulation of ERK and not JNK or p38 was responsible. Given the role that Th17 cells play in autoimmunity, the susceptibility of the reconstituted animals was also assessed using the MOG-induced model of EAE and consistent with the functional Th17 deficit, these animals were less susceptible to disease indicating an essential role for this MKP in autoimmune responses [198].

Finally, a recent study has explored the reason for the embryonic/prenatal lethality in mice lacking *DUSP16*/MKP-7 and revealed an essential role for this MKP in brain development. Embryos lacking *DUSP16*/MKP-7 exhibited congenital obstructive hydrocephalus together with brain overgrowth. This was secondary to blockage of the midbrain aqueduct by the expansion of neural progenitor cells, eventually preventing the outflow of cerebrospinal fluid. Interestingly only an increase in cells staining positively for phospho-p38 was observed in the affected regions of the CNS indicating that this MAPK, rather than ERK or JNK could be the relevant target [199].

Taken together these studies reveal the first essential role for a member of the MKP family of enzymes in brain development and as the phenotype of *DUSP16*^−/−^ mice recapitulates aspects of different human neurodevelopmental disorders suggests that either *DUSP16*/MKP-7 or the pathways it regulates may be implicated. They also reveal specific defects in innate and adaptive immunity in mice lacking *DUSP16*/MKP-7. In particular the specific role of *DUSP16*/MKP-7 in promoting autoimmunity make it a potential therapeutic target in a range of human disorders such as inflammatory bowel disease, rheumatoid arthritis and lupus.

## 5 Conclusions and future perspectives

The past decade has seen an acceleration in the use of GEM models to probe the physiological and pathophysiological roles of the MKPs and these have provided a wealth of information for certain members of the family, particularly in relation to the functional regulation of the immune system, but also in metabolic disease and cancer. As is the case for other classes of protein phosphatases, it is abundantly clear that MKPs are not merely passive “erasers” of protein phosphorylation, but instead form a complex network of activities in cells and tissues that act to regulate the spatiotemporal activity of the different MAP kinase pathways and play essential roles in regulating key physiological outcomes.

Several themes have emerged, one of which is the importance of compartmentalised regulation of MAPK signalling by activities in the nucleus and cytoplasm as exemplified by the regulation of nuclear JNK activity by *DUSP1*/MKP-1 in metabolic control [50] and nuclear ERK activity by DUSP5 in cancer [128]. It is also clear that there are both tissue and cell type specific differences in the MAPK isoforms targeted by particular MKPs, one example being the preference of *DUSP1*/MKP-1 for inactivation of p38 MAPK in macrophages and dendritic cells [30,35] whereas JNK is the relevant substrate in T cells [33]. Thus far, we do not have any detailed grasp of how these specificities may be altered *in vivo*. This must be addressed in future work as must the validity of putative non-MAPK substrates for certain MKPs invoked in disease models. The latter include the possibility that STAT3 is directly targeted by *DUSP2* in innate immunity [87], the potential regulation of FOXO1 and as yet uncharacterised non MAPK substrates by *DUSP6*/MKP-3 in metabolic regulation [154] and endothelial inflammation [151] respectively and modulation of PKC activity by *DUSP7*/MKP-X in oocyte meiotic progression [173].

Finally, there are numerous examples where knockout phenotypes indicate that certain MKPs may be therapeutic targets in human disease. These include inhibition of *DUSP1*/MKP-1 in combatting obesity and depression [50,73] and *DUSP2* as a potential anti-inflammatory drug target [86]. However, caution must be exercised here as inhibition of *DUSP1*/MKP-1 could also result in more severe inflammatory responses and many existing anti-inflammatory agents, such as glucocorticoids, actually enhance the expression and activity of this MKP as part of their mode of action. Clearly specificity of action towards individual MKPs would also be crucial in any strategy to target these enzymes and the avoidance of undesirable side effects. In this regard and due to the involvement of a redox active cysteine residue in catalysis, the PTPase superfamily has long been regarded as “undruggable”. However, recent progress in the development of allosteric PTPase inhibitors [200,201] gives hope that future work will lead to the development of highly specific small molecules able to target MKP activity, which will then allow a meaningful exploration of their therapeutic potential.

## Transparency document

Transparency document. Image 1
